# Physiological comparison of high-flow oxygen via endotracheal tube and T-piece strategies during spontaneous breathing trials: a randomized crossover study

**DOI:** 10.1186/s13054-026-06266-5

**Published:** 2026-08-18

**Authors:** Shan-Shan Xu, Rui-Zhi Zhang, Jun-Tong Liu, Lin Wang, Meng-Xue Hou, Xu An, Ming-Yue Miao, Hong-Liang Li, Yue-Fu Wang, Jian-Xin Zhou

**Affiliations:** 1 Emergency and Critical Care Medical Center, Beijing Shijitan Hospital, Capital Medical University, Beijing, China; 2 Clinical and Research Center on Acute Lung Injury, Capital Medical University, Beijing, China; 3 Department of Critical Care Medicine, Beijing Fengtai You’anmen Hospital, Beijing, China

**Keywords:** Spontaneous breathing trial, High-flow oxygen via endotracheal tube, Electrical impedance tomography, Esophageal pressure

## Abstract

**Background:**

The optimal spontaneous breathing trial (SBT) strategy remains uncertain because commonly used approaches involve distinct physiological trade-offs. Pressure support ventilation (PSV) may reduce inspiratory effort, whereas T-piece trials more closely approximate unsupported breathing after extubation. Neither approach has shown clear superiority for extubation outcomes. High-flow oxygen (HFO) delivered via endotracheal tube has been proposed as an alternative SBT strategy, but the physiological effects remain insufficiently defined.

**Methods:**

In a randomized crossover physiological study, patients receiving mechanical ventilation for at least 24 h and deemed ready for weaning underwent five SBT conditions: T-piece and four HFO via endotracheal tube settings combining two interfaces with different expiratory port diameters (9.8 and 6.9 mm) and two flow rates (40 and 60 L/min). Airway and esophageal pressures, respiratory variables, and electrical impedance tomography parameters were measured during baseline mechanical ventilation and under each SBT condition. Esophageal pressure was also measured after extubation for comparison with post-extubation inspiratory effort. A complementary bench study assessed 24 simulated conditions across four interface diameters and six flow rates.

**Results:**

Twenty patients were analyzed. Compared with T-piece, HFO-6.9 at 60 L/min produced the largest increases in mean expiratory airway pressure (median paired difference, 7.0 cm H_2_O [95% CI, 6.7–7.4]) and PEEP (6.0 cm H_2_O [5.8–6.1]). HFO-6.9 at 60 L/min also attenuated global end-expiratory lung volume loss (321 mL [267–523]), reduced dynamic transpulmonary driving pressure (3.3 cm H_2_O [1.8–4.1]), improved PaO_2_/FiO_2_ (28 mmHg [14–52]), and reduced respiratory rate (2 breaths/min [1–4]) compared with T-piece. No statistically significant difference in ΔPes was observed across the five SBT conditions and post-extubation measurements (*p* = 0.24). Bench simulations supported the flow- and diameter-dependent pressure effects.

**Conclusions:**

During SBT, HFO via endotracheal tube generated flow- and expiratory port diameter-dependent increases in airway pressure and lung volume, with lower dynamic transpulmonary driving pressure and improved oxygenation; no statistically significant difference in ΔPes was observed across HFO, T-piece, and post-extubation measurements. These findings support tracheal HFO as a physiologically distinct SBT strategy that may inform individualized SBT selection. Its relationship to clinical outcomes requires further evaluation.

**Trial registration:**

ClinicalTrials.gov (NCT06816706). Registered on 6 February 2025.

**Supplementary Information:**

The online version contains supplementary material available at https://doi.org/10.1186/s13054-026-06266-5.

## Introduction

The spontaneous breathing trial (SBT) is considered a key step during the weaning process from mechanical ventilation. Because both premature and delayed extubation are associated with adverse outcomes, SBT is widely used to assess a patient’s ability to sustain spontaneous breathing after extubation [1]. Current clinical practice guidelines recommend routinely performing an SBT before extubation [2, 3].

The two most commonly applied SBT strategies are T-piece and low-level pressure support ventilation (PSV), which have distinct physiological effects [1, 4]. Low-level PSV can reduce inspiratory effort during the trial and may underestimate the respiratory load encountered after extubation. In contrast, T-piece trials more closely approximate unsupported breathing after withdrawal of ventilatory assistance [5–8]. However, recent randomized trials and the clinical practice guidelines suggest broadly similar extubation outcomes between PSV and T-piece trials, with either approach considered acceptable in the general population of mechanically ventilated patients [3, 9, 10]. Because each method represents a different balance between providing support and testing unsupported breathing, the preferred SBT approach may differ across patient populations [3, 8]. These uncertainties have motivated interest in alternative SBT strategies, with further study needed to define their physiological effects and clinical roles.

High-flow oxygen (HFO) via endotracheal tube has recently been proposed as a potential SBT approach [11–14]. However, available clinical studies have yielded mixed or inconclusive results regarding extubation-related outcomes, leaving the clinical role of this strategy uncertain [11–13, 15]. Interpreting these clinical findings requires a more precise understanding of the physiological effects of tracheal HFO during the trial: whether it unloads the respiratory muscles, preserves lung volume, modifies regional ventilation, or simply changes oxygen delivery. Clarifying these mechanisms is essential for defining the patients and settings in which this strategy might be physiologically useful.

In this randomized crossover study, we compared the physiological effects of tracheal HFO delivered through interfaces with different expiratory port diameters and flow settings with T-piece during SBT. A complementary bench experiment was performed to clarify the mechanical effects of expiratory port diameter and flow rate. We hypothesized that HFO via endotracheal tube would generate flow- and interface-dependent physiological effects that may define a distinct physiological condition between unsupported T-piece breathing and PSV SBT.

## Methods

### Clinical study

#### Study design and settings

This randomized crossover clinical study was conducted in four general intensive care units (ICUs) of two hospitals from May 2025 to December 2025 (Beijing Shijitan Hospital, Capital Medical University, Beijing, China and Beijing Fengtai You’anmen Hospital, Beijing, China). The Institutional Ethics Committees of both hospitals approved the study protocol (IIT2024-157-002 and KY-202505-01). The study was registered at ClinicalTrials.gov (NCT06816706). Written informed consent was obtained from all enrolled patients or their legal surrogates. The protocol and statistical analysis plan have been previously published [16].

#### Patients

Adult patients receiving invasive mechanical ventilation for at least 24 h who were deemed ready for weaning were screened for inclusion. The readiness for weaning was evaluated following the currently recommended clinical guidelines [2].

Exclusion criteria included: 1) age younger than 18 years; 2) pregnancy; 3) hemodynamic instability; 4) respiratory and oxygenation instability; 5) medical history of neuromuscular diseases or phrenic nerve injury; 6) contraindications for esophageal pressure catheter insertion; 7) evidence of active air leak from the lung; 8) contraindications to electrical impedance tomography (EIT) monitoring; and 9) anticipated withdrawal of life support (details available in Supplementary Material 1).

#### Study procedures

After enrollment, an esophageal balloon catheter was placed in the esophagus, with proper positioning validated by the Baydur maneuver [17, 18]. Airflow, airway pressure, and esophageal pressure were continuously collected via a heated Fleisch pneumotachograph and pressure transducers. All signals were recorded simultaneously at 200 Hz for offline analysis (ICU–Lab 2.5 Software Package, KleisTEK Engineering, Bari, Italy). An EIT-dedicated belt containing 16 electrodes was positioned around the patient’s thorax at the fifth or sixth intercostal space and connected to the EIT monitor (PulmoVista 500; Dräger Medical, Germany). EIT data were captured at 20 Hz and analyzed offline via dedicated software (Dräger EIT Data Analysis Tool 6.3, Lübeck, Germany).

Airway secretions were cleared as clinically indicated before recordings to minimize interference with waveform acquisition. Flow and airway pressure waveforms were continuously inspected, with suctioning during the washout period performed only when secretion-related artifacts, such as sawtooth oscillations, were observed.

A 10-min baseline recording was obtained using the PSV and positive end-expiratory pressure (PEEP) levels set by the responsible physician prior to inclusion. Then, each patient received five 15-min SBTs in a computer-generated random order:

- T-piece (6 L/min);
- HFO via endotracheal tube with a 9.8 mm expiratory port (HFO-9.8) at 40 L/min;
- HFO-9.8 at 60 L/min;
- HFO via endotracheal tube with a 6.9 mm expiratory port (HFO-6.9) at 40 L/min;
- HFO-6.9 at 60 L/min.

The randomized treatment sequence for each patient is provided in Supplementary Material 1, Table S1. A 10-min washout period with ventilator reconnection to baseline settings was implemented between phases. Each 15-min period was designed as a study-specific experimental physiological phase to allow physiological stabilization and repeated measurements, with reference to previous research on SBT strategies [3, 7, 8]. This design kept the total protocol duration within approximately 2 h, reducing the risk of fatigue-related fluctuations in physiological parameters.

Humidified T-piece oxygen was delivered using an air entrainment nebulizer and T-tube. HFO via endotracheal tube was delivered via the Aeonmed NeoHiF-i7 device (Beijing Aeonmed Company, Beijing, China) with the interface positioned between the endotracheal tube and the breathing circuit. Two interfaces were evaluated, with expiratory port diameters of 6.9 mm and 9.8 mm (Xiangyue Medical Ltd, Tianjin, China), defined as the minimal internal diameter at the expiratory port (Supplementary Material 1, Figure S1). For all SBT phases with tracheal HFO, FiO_2_ was maintained at 0.4 and temperature at 37 °C.

Vital signs, end-tidal carbon dioxide pressure (EtCO_2_) and respiratory mechanics including flow, airway pressure, esophageal pressure, and EIT data were collected during each phase (Supplementary Material 1, Figure S2). Arterial blood gas analyses were performed at the end of the following phases: T-piece, HFO-9.8 and HFO-6.9 at 60 L/min.

After completing all study procedures, participants were reconnected to the ventilator. The extubation was decided by the attending physicians, in accordance with guidelines and their clinical judgment [2].

Supplemental oxygen after extubation was determined at the clinicians’ discretion. Esophageal pressure data were collected for 3 min after extubation when the patient’s breathing condition remained stable. Details of study procedures are shown in Supplementary Material 1.

#### Data collection and analysis

Patient demographics and clinical characteristics were collected at study entry.

Respiratory data were analyzed from ten consecutive stable breaths recorded during the final three minutes of each study phase [7, 8]. Details of data analysis are shown in Supplementary Material 1.

Mean and peak inspiratory and expiratory airway pressure, and PEEP were recorded from the airway pressure signal. Tidal volume (V_T_), respiratory rate, and minute ventilation were recorded from the flow signal. The dynamic transpulmonary driving pressure (∆P_L,dyn_) was calculated as the difference between the maximum transpulmonary pressure (airway pressure minus esophageal pressure) and the value at the onset of inspiratory effort, identified as the point of negative deflection of esophageal pressure with a rapid change in slope [18]. Inspiratory and expiratory airway resistances were derived at a standardized flow rate of 0.2 L/s [19].

The tidal swing of esophageal pressure during inspiration (∆Pes) was defined as the maximal drop in esophageal pressure from the onset of inspiratory effort to the inspiratory nadir. To quantify inspiratory workload, the esophageal pressure–time product per minute (PTPes) was computed as the time integral of the area between the esophageal pressure curve and the estimated chest wall recoil pressure from the onset of inspiratory effort to the end of inspiration, identified by zero flow, multiplied by the respiratory rate. Chest wall compliance was estimated as 4% of the predicted vital capacity, calculated according to age and height [20]. The estimated chest wall recoil pressure was calculated as volume curve divided by chest wall compliance. The pressure generated by respiratory muscles during inspiration (Pmus) was determined as the peak difference between esophageal pressure and chest wall recoil pressure. Total, elastic, and resistive work of breathing (WOB, WOB_ela_, and WOB_res_) were integrated utilizing the Campbell diagram framework [21, 22].

EIT data were analyzed to track regional gas distribution and lung volume variations. Global and regional changes in end-expiratory lung volume (∆EELV_glob_, ∆EELV_dep_, and ∆EELV_non-dep_) during each SBT phase were calculated relative to the PSV baseline recorded before the five randomized SBT phases. End-expiratory impedance shifts were converted to volume changes using an individual calibration ratio (mL/∆Z), which was derived from simultaneous tidal volume and impedance changes during stable breathing immediately before an end-expiratory occlusion at the PSV baseline [12, 23, 24]. Furthermore, the dependent fraction of ventilation, regional tidal ventilation distribution in dependent and non-dependent lung regions (V_Tdep_, and V_Tnon-dep_), center of ventilation (COV), and global inhomogeneity (GI) index were calculated to characterize lung homogeneity and ventilation distribution [25–27].

All patients were followed up until their hospital discharge, death, or 28 days post-enrollment, whichever occurred first. The weaning and extubation outcomes, post-extubation management and length of stay were collected.

#### Study endpoints

The primary endpoints were the mean expiratory airway pressure, PEEP, and ∆EELV. The secondary endpoints included the following physiological parameters: V_T_, respiratory rate, ∆Pes, PTPes, Pmus, ∆P_L,dyn_, EtCO_2_, and SpO_2_.

### Bench experiment

An 8.0-mm endotracheal tube was connected to an active lung simulator (ASL 5000, IngMar Medical, USA) to mimic spontaneous breathing. A total of 24 simulated breathing conditions were generated based on previous bench and clinical studies to represent a broad range of respiratory mechanics during SBTs, constructed by a factorial combination of two compliance levels (40 and 60 mL/cm H_2_O), two airway resistance levels (10 and 20 cm H_2_O s/L), three levels of inspiratory effort (Pmus: − 5, − 10, and − 15 cm H_2_O), and two respiratory rates (20 and 30 breaths/min), which mapped onto normal, ARDS-like, and COPD-like clinical phenotypes [10, 28, 29].

We evaluated four tracheal HFO interfaces with expiratory port diameters of 6.9 mm (HFO-6.9), 8.6 mm (HFO-8.6; Veoflo, Flexicare, Mountain Ash, United Kingdom), 9.8 mm (HFO-9.8), and 14.6 mm (HFO-14.6; OPT970 Optiflow+; Fisher & Paykel, Auckland, New Zealand), including the two interfaces used in the clinical study (Supplementary Material 1, Figure S3). Each simulation condition was tested using the four interfaces at six flow settings (10–60 L/min), with FiO_2_ set at 0.21 and temperature maintained at 37 °C, yielding a total of 576 measurement segments. Flow and airway pressure were recorded through ASL software 3.6 at 512 Hz, with the last 10 breaths of each 5-min period used for analysis. The primary outcomes of the bench study were the mean expiratory airway pressure and PEEP. The secondary outcomes included the following physiological parameters: ∆EELV, ∆P_L,dyn_, V_T_, peak flow, and airway resistance. Detailed methodological descriptions are provided in Supplementary Material 1.

### Statistical analysis

Continuous variables are presented as median with interquartile range, and categorical variables are reported as counts and percentages. Given the small sample size and physiological crossover design, comparisons across the five SBT conditions and post-extubation phase were primarily performed using the Friedman test, with Dunn’s test and Bonferroni correction for post hoc pairwise comparisons. For the most important physiological outcomes, absolute paired differences were reported as median paired differences with 95% bootstrap confidence intervals (CIs). For ΔPes, paired differences versus post-extubation measurements were additionally reported. To assess robustness to potential period/order and carryover effects, post hoc mixed-effects sensitivity analyses were performed for the prespecified primary and secondary endpoints measured across the five randomized SBT conditions. Patient was included as a random intercept, and treatment condition, study period/order, and preceding treatment condition were included as fixed effects. Global P values for fixed effects were obtained using Type III ANOVA with Satterthwaite’s method for degrees of freedom. Pairwise P values for comparisons versus T-piece were obtained from estimated marginal means, with Holm adjustment for multiple comparisons. Exploratory descriptive subgroup plots were generated according to ICU admission category to illustrate potential heterogeneity in physiological responses. These plots were not intended for formal subgroup hypothesis testing because of the small subgroup sizes.

For the sample size calculation, we employed the repeated-measures ANOVA method using mean expiratory airway pressure, PEEP, and ΔEELV as multiple co-primary endpoints. Because previous studies of HFO via endotracheal tube focused mainly on clinical outcomes rather than these physiological variables, no endpoint-specific reference data were available [11–13]. We therefore adopted a Cohen-based medium effect size of 0.25 alongside a statistical power of 0.80 and a significance level set at 0.05 to achieve adequate statistical power [30]. This yielded a required sample size of 20 subjects, calculated using G*Power version 3.1.9.7, and was intended to provide adequate power to detect a medium within-patient physiological effect in this crossover design. Patients who were unable to complete all study phases were excluded. Additional patients were recruited to ensure that the final analysis included 20 subjects with complete SBTs and post-extubation datasets.

In the bench experiment, continuous variables are presented as mean ± SD. A linear mixed-effects model was used to evaluate the effects of the HFO via endotracheal tube interface diameter, flow rate (categorical factors), and their interaction (fixed effects). The 24 simulated scenarios were modeled as random intercepts to account for variability across simulated phenotypes. Model-estimated marginal means with 95% CIs were derived for graphical presentation.

Statistical analysis was performed using IBM SPSS Statistics 26.0, GraphPad Prism 9.0, and R (version 4.5.2) software. A *p*-value of less than 0.05 was considered statistically significant.

## Results

### Clinical study

**Fig. 1:**
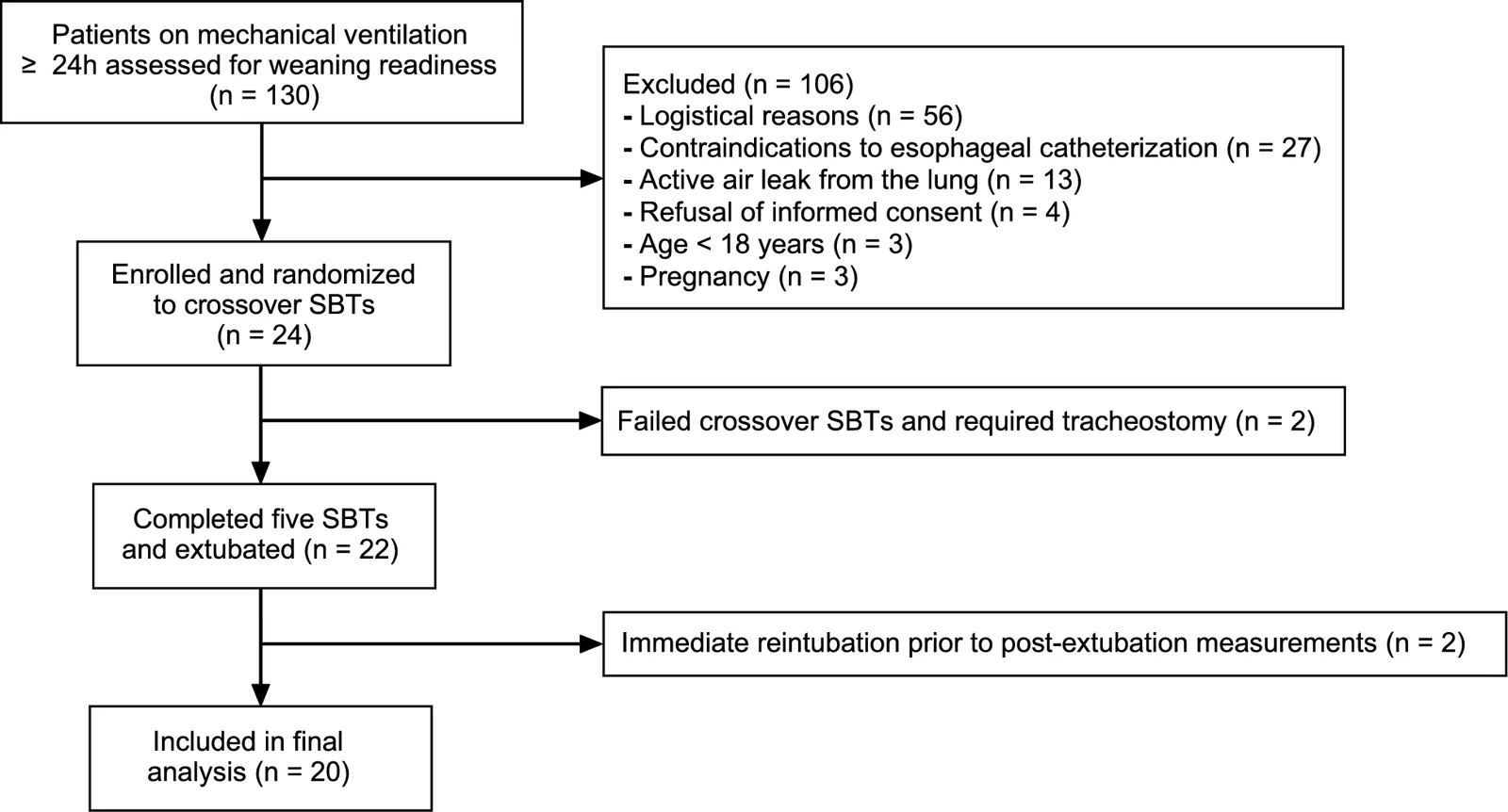
Flowchart of the study. SBT: spontaneous breathing trial

Between May and December 2025, 130 mechanically ventilated patients meeting weaning readiness criteria were screened across participating ICUs. Twenty-four patients were enrolled; two failed crossover SBTs and underwent tracheostomy, and two were reintubated immediately after extubation with the subsequent loss of post-extubation data. Thus, 20 patients were included in the final analysis (Fig. 1).

Baseline characteristics are shown in Table 1. Before enrollment, the median duration of mechanical ventilation was 6 (5–10) days. At baseline, the median pressure support was 8 (6–8) cm H_2_O and PEEP was 5 (5–6) cm H_2_O. The median interval from SBT completion to extubation was 24 (2–24) hours.

**Table 1:** Baseline characteristics of enrolled patients

**Baseline characteristics**	**Patients (n=20)**
Age, years	66 (51–79)
Male, n (%)	10 (50%)
Body mass index, kg/m^2^	23 (20–27)
APACHE II at ICU admission	16 (10–18)
Richmond Agitation Sedation Scale	0 (0–0)
Main reason for ICU admission, n (%) Septic shock Pneumonia Ischemic stroke Hemorrhagic stroke	8 (40%) 8 (40%) 2 (10%) 2 (10%)
Endotracheal tube internal diameter, mm, n (%)	
7.0 7.5 8.0	5 (25%) 11 (55%) 4 (20%)
Baseline ventilation parameters	
Pressure support, cm H_2_O PEEP, cm H_2_O	8 (6–8) 5 (5–6)
FiO_2_ of T-piece SBT, (%) Duration of mechanical ventilation, days	39 (37–43) 7 (5–11)
Time from SBT completion to extubation, hours	24 (2–24)
Extubation failure, n (%)	1 (5%)
Post extubation oxygen management, n (%) Noninvasive ventilation Nasal HFO Standard oxygenation	3 (15%) 14 (70%) 3 (15%)
Length of ICU stay, days	13 (9–17)
In-ICU mortality, n (%)	2 (10%)

APACHE II: Acute Physiology and Chronic Health Evaluation II; ICU: intensive care unit; PEEP: positive end-expiratory pressure; SBT: spontaneous breathing trial; HFO: high-flow oxygen

Data are reported as median (interquartile range) or number (percentage)

#### Airway pressure and lung volume

**Fig. 2:**
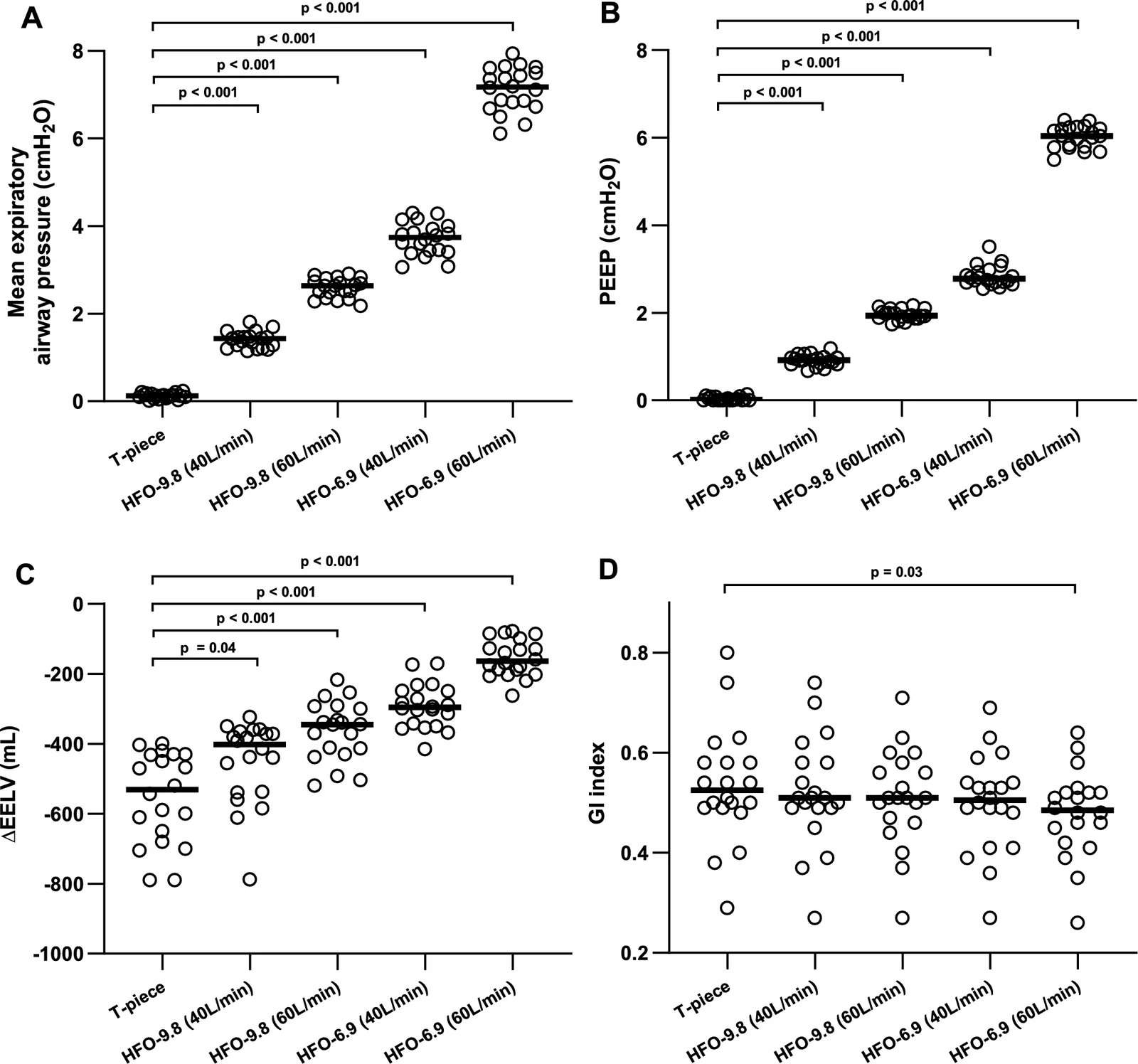
Airway pressure and ventilation distribution across different study phases. Individual patient values and medians (horizontal lines) of (**A**) mean expiratory airway pressure, (**B**) positive end-expiratory pressure (PEEP), (**C**) change of end-expiratory lung volume (ΔEELV), and (**D**) global inhomogeneity (GI) index across the study phases. Overall comparisons were performed using the Friedman test. Significant pairwise comparisons between each HFO condition and T-piece are indicated in the figure. P values were adjusted using Dunn’s test with Bonferroni correction. HFO-6.9: HFO via endotracheal tube with a 6.9 mm expiratory port; HFO-9.8: HFO via endotracheal tube with a 9.8 mm expiratory port

Airway pressure and lung volume variables across the five study phases and PSV baseline are shown in Table 2, Table S2 (Supplementary Material 1), Table S4 (Supplementary Material 1) and Fig. 2. Airway pressure parameters differed among the five SBT study phases (all *p*<0.001). Compared with T-piece, all tracheal HFO settings increased inspiratory and expiratory peak and mean airway pressure (all *p* < 0.001).

**Table 2:** Airway pressure, lung volume and gas exchange across the five study phases

	**T–piece**	**HFO−9.8** **(40 L/min)**	**HFO−9.8** **(60 L/min)**	**HFO−6.9** **(40 L/min)**	**HFO−6.9** **(60 L/min)**	***p-*value**
Airway pressure						
PEEP, cm H_2_O	0.0 (0.0–0.1)	0.9 (0.8–1.0) ^*^	1.9 (1.9–2.1) ^*^	2.8 (2.7–3.0)^*^	6.0 (5.8–6.2)^*^	< 0.001
Peak inspiratory airway pressure, cm H_2_O	−0.3 (− 0.5–−0.2)	−0.1 (− 0.3–0.1) ^*^	0.5 (0.2–0.8) ^*^	0.3 (0.0–0.7) ^*^	2.5 (1.8–2.8) ^*^	< 0.001
Mean inspiratory airway pressure, cm H_2_O	−0.1 (− 0.3–−0.1)	0.2 (0.1–0.4) ^*^	1.0 (0.8–1.2) ^*^	1.0 (0.8–1.3) ^*^	3.5 (3.0–3.6) ^*^	< 0.001
Peak expiratory airway pressure, cm H_2_O	0.3 (0.2–0.3)	1.9 (1.8–2.1) ^*^	3.2 (3.0–3.6) ^*^	4.4 (4.1–4.7) ^*^	8.0 (7.5–8.1) ^*^	< 0.001
Mean expiratory airway pressure, cm H_2_O	0.1 (0.1–0.2)	1.4 (1.2–1.5) ^*^	2.6 (2.4–2.8) ^*^	3.7 (3.4–4.0) ^*^	7.2 (6.8–7.6) ^*^	< 0.001
Lung volume and homogeneity						
V_Tdep_, mL	186 (138–289)	183 (134–257)	191 (126–281)	192 (126–265)	216 (131–284)	0.35
V_Tnon–dep_, mL	205 (183–279)	221 (181–305)	206 (172–304)	209 (156–254)	205 (154–254)	0.004
∆EELV_glob_, mL	−532 (− 673–−431)	−402 (− 539–−367)^*^	−345 (− 426–−295)^*^	−296 (− 348–−249)^*^	−164 (− 199–−106)^*^	< 0.001
∆EELV_dep_, mL	−224 (− 390–−185)	−197 (− 314–−140)^*^	−163 (− 243–−128)^*^	−155 (− 189–−97)^*^	−62 (− 102–−43)^*^	< 0.001
∆EELV_non–dep_, mL	−285 (− 334–−212)	−231 (− 265–−182)^*^	−185 (− 228–−144)^*^	−146 (− 189–−101)^*^	−79 (− 109–−42)^*^	< 0.001
Dependent fraction of ventilation, %	46 (38–55)	46 (34–56)	46 (33–58)	53 (37–60)^*^	53 (35–60) ^*^	0.001
COV, %	48 (44–51)	47 (43–51)	48 (44–53)	48 (44–51)	48 (44–53)	0.17
GI index	0.53 (0.49–0.58)	0.51 (0.49–0.58)	0.51 (0.46–0.58)	0.51 (0.43–0.54)	0.49 (0.43–0.52) ^*^	0.003
Gas exchange						
pH	7.43 (7.42–7.44)	–	7.42 (7.41–7.45)	–	7.42 (7.40–7.45)	0.14
PaO_2_, mm Hg	105 (86–121)	–	109 (92–120)	–	121 (102–142) ^*^	0.001
PaCO_2_, mm Hg	41 (38–43)	–	40 (38–44)	–	41 (38–45)	0.11
PaO_2_/FiO_2_, mm Hg	268 (219–316)	–	273 (229–299)	–	303 (256–354) ^*^	<0.001

HFO-9.8: HFO via endotracheal tube with a 9.8 mm expiratory port; HFO-6.9: HFO via endotracheal tube with a 6.9 mm expiratory port; PEEP: positive end-expiratory pressure; V_Tdep_: tidal volume distending dependent regions; V_Tnon-dep_: tidal volume distending nondependent regions; ∆EELV_glob_: global change of end-expiratory lung volume; ∆EELV_dep_: change of end-expiratory lung volume in dependent regions; ∆EELV_non–dep_: change of end-expiratory lung volume in nondependent regions; COV: center of ventilation; GI index: global inhomogeneity index

Data are presented as median (interquartile range). *p*-values represent global differences across the five conditions determined by the Friedman test. Post hoc pairwise comparisons were performed using Dunn’s test with Bonferroni correction

^*^p < 0.05 versus T-piece. Full pairwise comparisons are provided in Table S2 (Supplementary Material 1)

During T-piece SBT, airway pressure remained < 0.5 cm H_2_O throughout the respiratory cycle. The increase in airway pressure in tracheal HFO was flow- and expiratory port diameter-dependent. Compared with T-piece, HFO-6.9 at 60 L/min produced the largest increases in mean expiratory airway pressure (median paired difference, 7.0 cm H_2_O [95% bootstrap CI, 6.7–7.4 cm H_2_O]) and PEEP (median paired difference, 6.0 cm H_2_O [95% bootstrap CI, 5.8–6.1 cm H_2_O]). At the same flow, airway pressure was higher with HFO-6.9 than with HFO-9.8, whereas increasing flow from 40 to 60 L/min further increased airway pressure for both expiratory port diameters.

∆EELV, GI, and the dependent fraction of ventilation differed across phases (*p* < 0.001, *p* = 0.003, and *p* = 0.001, respectively), whereas COV did not. Compared with T-piece, all HFO SBTs attenuated the loss of global, dependent, and nondependent ∆EELV (all *p* < 0.001). The largest effect was observed with HFO-6.9 at 60 L/min, which attenuated the loss of ∆EELV_glob_ versus T-piece by a median paired difference of 321 mL (95% bootstrap CI, 267–523 mL). HFO-6.9 increased dependent ventilation at both 40 and 60 L/min (*p* = 0.015 and *p* < 0.001), whereas HFO-9.8 had no significant effect. GI decreased only with HFO-6.9 at 60 L/min (*p* = 0.03).

#### Inspiratory effort

No statistically significant difference in ΔPes was observed across T-piece, the four tracheal HFO conditions, and post-extubation measurements (*p* = 0.24), with median values of 15 (11–16), 14 (10–17), 13 (10–17), 14 (10–17), 13 (10–16), and 15 (11–17) cm H_2_O, respectively. Paired HFO–post-extubation differences were small in magnitude, ranging from − 1.2 to − 0.6 cm H_2_O, and are reported with 95% CIs in Table S5. Across the five study conditions, ΔPes, PTPes, and Pmus did not differ. In contrast, baseline PSV was associated with significantly lower inspiratory effort compared with all five study conditions and the post-extubation phase (Table 3, Tables S3–S5 in Supplementary Material 1, Fig. 3).

**Table 3:** Hemodynamics, respiratory variables and inspiratory effort across the five study phases

	**T–piece**	**HFO−9.8** **(40 L/min)**	**HFO−9.8** **(60 L/min)**	**HFO−6.9** **(40 L/min)**	**HFO−6.9** **(60 L/min)**	***p-*value**
Hemodynamics						
Heart rate, beats/min	89 (79–101)	90 (75–104)	90 (78–103)	92 (79–105)	91 (77–103)	0.80
Mean blood pressure, mm Hg	95 (85–104)	92 (85–105)	94 (87–104)	93 (86–105)	95 (88–106)	0.42
SpO_2_, %	99 (97–100)	99 (98–100)	99 (98–100)	99 (98–100)	99 (98–100)	0.36
Respiratory variables						
Respiratory rate, breaths/min	22 (16–27)	20 (16–26)	20 (16–27)	20 (16–25)^*^	19 (16–24)^*^	0.002
V_T_, mL	421 (339–530)	438 (353–558)	423 (331–553)	423 (321–530)	429 (342–523)	0.22
Minute ventilation, L/min	9.0 (7.2–10.4)	8.7 (6.6–9.9)	8.8 (6.3–9.7)	8.5 (6.4–9.3)^*^	8.3 (6.1–9.5) ^*^	< 0.001
EtCO_2_, mm Hg	39 (32–43)	39 (32–43)	39 (32–43)	39 (32–43)	39 (32–43)	0.11
Expiratory time, s	1.66 (1.43–2.56)	1.91 (1.45–2.57)	1.85 (1.42–2.59)	1.74 (1.53–2.72)	1.98 (1.64–2.46)	0.03
Expiratory time/ total respiratory time, %	64 (62–70)	65 (62–71)	65 (62–71)	65 (63–70)	67 (62–70)	0.11
Peak inspiratory flow, L/s	0.63 (0.49–0.80)	0.62 (0.50–0.73)	0.61 (0.48–0.78)	0.60 (0.45–0.71)	0.57 (0.46–0.70)^*^	0.005
Peak expiratory flow, L/s	0.42 (0.38–0.50)	0.41 (0.35–0.45)	0.40 (0.32–0.43) ^*^	0.35 (0.31–0.42) ^*^	0.32 (0.27–0.37) ^*^	< 0.001
Inspiratory airway resistance, cm H₂O s/L	5.1 (3.8–7.3)	6.5 (4.4–9.7)	7.1 (4.5–10.1)^*^	7.4 (5.4–11.6)^*^	7.9 (5.4–12.8)^*^	< 0.001
Expiratory airway resistance, cm H₂O s/L	6.5 (4.2–8.8)	7.8 (6.3–8.9)^*^	7.9 (6.4–9.7)^*^	9.6 (6.9–11.8) ^*^	10.3 (8.4–13.9)^*^	< 0.001
Inspiratory effort						
∆Pes, cm H_2_O	15 (11–16)	14 (10–17)	13 (10–17)	14 (10–17)	13 (10–16)	0.99
PTPes, cm H₂O s/min	207 (157–251)	207 (158–234)	212 (151–242)	211 (155–247)	214 (156–260)	0.31
Pmus, cm H_2_O	17 (14–20)	17 (13–21)	16 (13–20)	16 (13–21)	15 (12–21)	0.84
∆P_L,dyn_, cm H_2_O	14 (10–16)	13 (9–16)	12 (9–15)^*^	11 (8–14)^*^	9 (7–13)^*^	< 0.001
WOB, J/min	8.7 (7.4–11.5)	8.9 (7.2–11.0)	8.4 (6.8–12.1)	9.3 (7.2–10.7)	9.3 (6.7–10.4)	0.86
WOB_res_, J/min	3.9 (2.4–5.5)	3.8 (2.9–4.9)	3.9 (3.0–5.8)	4.2 (3.1–5.3)	4.1 (3.2–5.5)	0.40
WOB_ela_, J/min	5.1 (3.9–6.5)	4.9 (4.2–5.9)	5.0 (3.7–5.9)	4.9 (3.9–5.6)^*^	4.8 (3.7–5.4)^*^	0.007

HFO-9.8: HFO via endotracheal tube with a 9.8 mm expiratory port; HFO-6.9: HFO via endotracheal tube with a 6.9 mm expiratory port; V_T_: tidal volume; EtCO_2_: end-tidal carbon dioxide pressure; ∆Pes: tidal swing of esophageal pressure during inspiration; PTPes: esophageal pressure time product per minute; Pmus: pressure generated by respiratory muscle during inspiration; ∆P_L,dyn_: dynamic transpulmonary driving pressure; WOB: work of breathing; WOB_res_: resistive work of breathing; WOB_ela_: elastic work of breathing

Data are presented as median (interquartile range). p-values represent global differences across the five conditions determined by the Friedman test. Post hoc pairwise comparisons were performed using Dunn’s test with Bonferroni correction

^*^p < 0.05 versus T-piece. Full pairwise comparisons are provided in Table S3 (Supplementary Material 1)

**Fig. 3:**
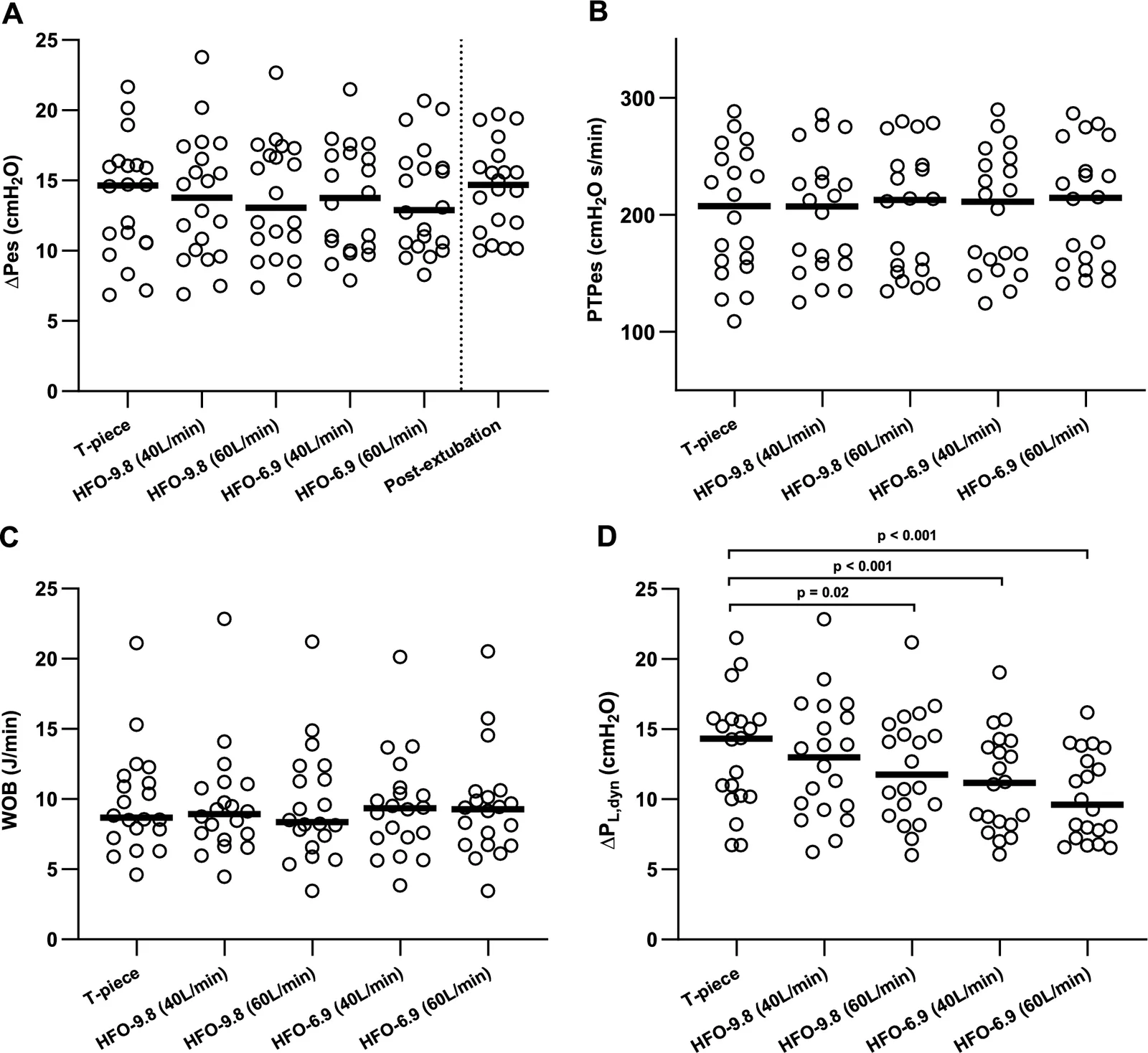
Inspiratory effort and lung stress across different study phases. Individual patient values and medians (horizontal lines) of (**A**) tidal swing of esophageal pressure during inspiration (∆Pes), (**B**) esophageal pressure–time product per minute (PTPes), (**C**) work of breathing (WOB), and (**D**) dynamic transpulmonary driving pressure (∆P_L,dyn_) across the study phases. Overall comparisons were performed using the Friedman test. Significant pairwise comparisons between each HFO condition and T-piece are indicated in the figure. P values were adjusted using Dunn’s test with Bonferroni correction. HFO-6.9: HFO via endotracheal tube with a 6.9 mm expiratory port; HFO-9.8: HFO via endotracheal tube with a 9.8 mm expiratory port

ΔP_L,dyn_ differed across SBT phases (*p* < 0.001). Compared with T-piece, HFO-9.8 at 60 L/min and HFO-6.9 at 40 and 60 L/min were associated with lower ΔP_L,dyn_ (*p* = 0.02, *p* < 0.001, and *p* < 0.001). The largest reduction occurred with HFO-6.9 at 60 L/min versus T-piece (median paired difference, 3.3 cm H_2_O [95% bootstrap CI, 1.8–4.1 cm H_2_O]) (Table 3, Table S3 in Supplementary Material 1, Fig. 3).

WOB and WOB_res_ did not differ among study phases, whereas WOB_ela_ varied (*p* = 0.007). Compared with T-piece, HFO-6.9 at 40 and 60 L/min was associated with reduced WOB_ela_ (*p* = 0.004 and *p* = 0.001).

#### Hemodynamics, respiratory variables and gas exchange

Baseline respiratory and hemodynamic variables did not differ before the five SBT phases. No hemodynamic differences were observed during SBT or after extubation (Table 3, Tables S4-S6 in Supplementary Material 1).

V_T_ and EtCO_2_ remained unchanged, whereas respiratory rate and minute ventilation varied (*p* = 0.002 and *p* < 0.001). Compared with T-piece, HFO-6.9 at 40 and 60 L/min reduced respiratory rate (*p* = 0.01 and *p* = 0.03) and slightly decreased minute ventilation (*p* = 0.04 and *p* = 0.03) without affecting EtCO_2_. The reduction in respiratory rate was greatest with HFO-6.9 at 60 L/min versus T-piece (median paired difference, 2 breaths/min [95% bootstrap CI, 1–4 breaths/min]). HFO-9.8 showed no differences versus T-piece (Table 3, Table S3 in Supplementary Material 1, Fig. 4).

**Fig. 4:**
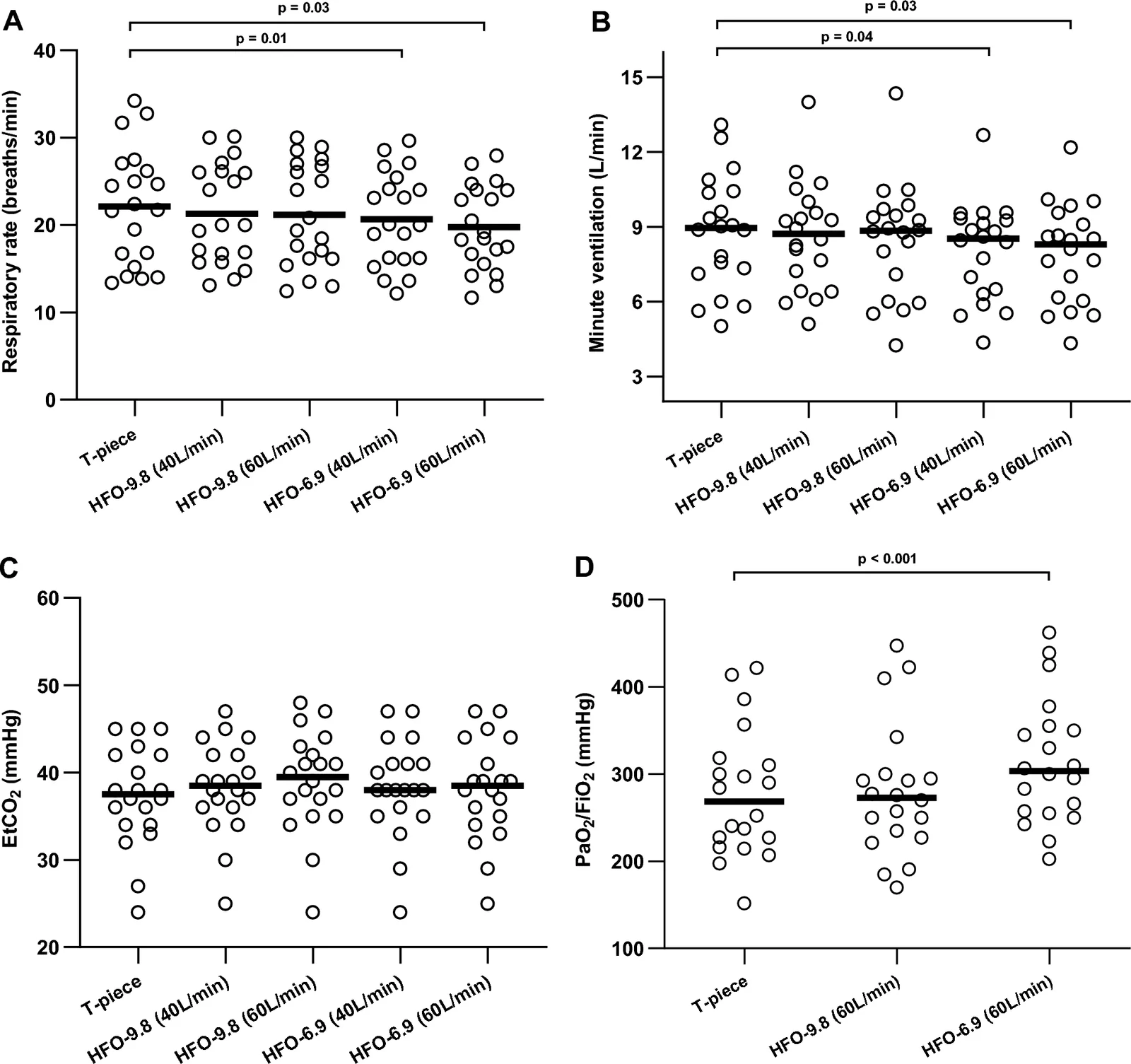
Respiratory rate, minute ventilation, and gas exchange across different study phases. Individual patient values and medians (horizontal lines) of (**A**) respiratory rate, (**B**) minute ventilation, (**C**) end-tidal carbon dioxide pressure (EtCO_2_), and (**D**) PaO_2_/FiO_2_ across the study phases. Overall comparisons were performed using the Friedman test. Significant pairwise comparisons between each HFO condition and T-piece are indicated in the figure. P values were adjusted using Dunn’s test with Bonferroni correction. HFO-6.9: HFO via endotracheal tube with a 6.9 mm expiratory port; HFO-9.8: HFO via endotracheal tube with a 9.8 mm expiratory port

Expiratory time varied slightly among study phases (*p* = 0.03), whereas its proportion within the respiratory cycle remained unchanged. Peak inspiratory and expiratory flow and airway resistance differed among SBT conditions. Compared with T-piece, HFO SBTs were associated with lower peak flows and higher airway resistance (all *p* < 0.05) (Table 3 and Table S3 in Supplementary Material 1).

Arterial oxygenation also differed across study phases (*p* = 0.001 for PaO_2_; *p* < 0.001 for PaO_2_/FiO_2_), whereas PaCO_2_ remained unchanged. Compared with T-piece, HFO-6.9 at 60L/min increased PaO_2_ (*p* = 0.007) and improved PaO_2_/FiO_2_ (*p* < 0.001; median paired difference, 28 mm Hg [95% bootstrap CI, 14–52]), whereas HFO-9.8 showed no significant differences (Table 2, Table S2 in Supplementary Material 1, Fig. 4).

Mixed-effects sensitivity analyses of the primary and secondary endpoints yielded results consistent with the primary Friedman analyses, with no evidence that period/order or preceding-treatment effects materially influenced the main conclusions (Supplementary Material 1, Table S7).

Exploratory descriptive plots stratified by admission category are provided in Figure S4 (Supplementary Material 1). Directional patterns in mean expiratory airway pressure, PEEP, ∆EELV, and ∆P_L,dyn_ appeared broadly similar across septic shock, pneumonia, and stroke subgroups, but subgroup sizes were small and no formal subgroup testing was performed.

### Bench experiment

Across simulated conditions, higher HFO flow rates and smaller expiratory port diameters were associated with reduced peak flow and V_T_, increased airway resistance, elevated airway pressure and PEEP, decreased transpulmonary pressure, and increased ΔEELV. Linear mixed-effects modeling demonstrated significant main and interaction effects of flow and interface diameter across all measured parameters (all *p* < 0.001) (Table S8 in Supplementary Material 1).

**Fig. 5:**
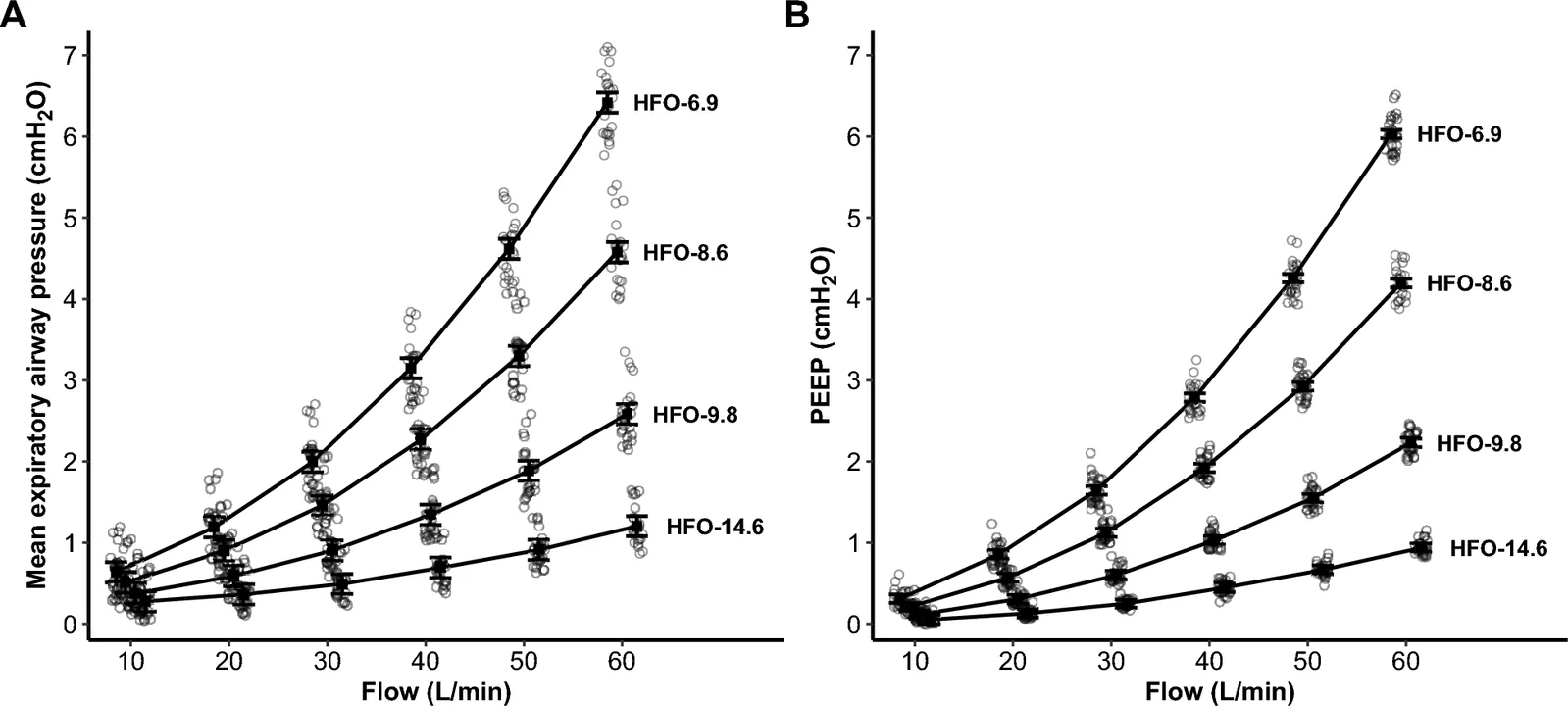
Effects of expiratory port diameter and flow rate in bench simulations. Expiratory port diameter and flow rate jointly determine (**A**) mean expiratory airway pressure and (**B**) positive end-expiratory pressure (PEEP). Circles represent individual simulated data points. Squares and connecting lines indicate model-estimated marginal means derived from linear mixed-effects modeling, with error bars representing 95% confidence intervals. Flow rate, diameter, and their interaction significantly influenced both variables (all *p*<0.001). HFO-6.9: HFO via endotracheal tube with a 6.9 mm expiratory port; HFO-8.6: HFO via endotracheal tube with an 8.6 mm expiratory port; HFO-9.8: HFO via endotracheal tube with a 9.8 mm expiratory port; HFO-14.6: HFO via endotracheal tube with a 14.6 mm expiratory port

PEEP and mean expiratory airway pressure increased progressively with increasing flow and decreasing expiratory port diameter (Fig. 5). Across most flow conditions, pressure levels in the bench experiment closely approximated those observed in patients.

## Discussion

This study compared the physiological effects of HFO via endotracheal tube and T-piece during SBT and explored mechanisms that may be relevant to ventilator liberation. The main findings were as follows. First, compared with T-piece, tracheal HFO increased airway pressure and end-expiratory lung volume, improved dependent ventilation and oxygenation, reduced ΔP_L,dyn_, and modestly reduced respiratory rate. Second, the downstream benefits in ventilation homogeneity and oxygenation occurred mainly with the smaller expiratory port at higher flows, suggesting a pressure threshold effect. Third, no statistically significant difference in ΔPes was observed across tracheal HFO, T-piece, and post-extubation measurements.

### Physiological mechanisms of tracheal HFO during SBT

Bench simulations and clinical measurements demonstrated a flow- and expiratory port diameter-dependent physiological cascade. Consistent with Poiseuille’s law, higher flows combined with smaller expiratory port diameters generated higher airway pressure [31]. This pressure effect was associated with stepwise increases in end-expiratory lung volume and redistribution of ventilation toward dependent lung regions. These changes were accompanied by improved ventilation homogeneity, lower ΔP_L,dyn_ and better oxygenation at similar FiO_2_, with modest reductions in respiratory rate and minute ventilation without a concomitant increase in PaCO_2_. The unchanged PaCO_2_ may suggest that alveolar ventilation was maintained despite lower minute ventilation, which may reflect improved ventilatory efficiency, although the underlying mechanism remains uncertain.

Nevertheless, not all bench observations translated directly to patients. Both bench and clinical data showed increased airway resistance and lower peak flow with smaller expiratory ports and higher set flow rates. In the bench model, fixed respiratory rate and timing meant that lower peak flow translated into lower V_T_. In patients, V_T_ remained largely unchanged, possibly because of physiological adaptation during tracheal HFO, including improved ventilation homogeneity, lower respiratory rate, and prolonged expiratory time.

This physiological cascade can be compared with other high-flow modalities. Nasal HFO generates flow-dependent airway pressure, increases end-expiratory lung volume, improves oxygenation, and reduces respiratory rate [23, 32, 33]. By contrast, HFO via tracheostomy, which bypasses upper-airway resistance, produces only small increases in airway pressure and no consistent rise in end-expiratory lung volume, partly because previously studied interfaces had large expiratory port diameters (14.6 mm) with limited expiratory resistance [34–36]. In the present study, HFO via endotracheal tube with the smaller 6.9 mm expiratory port generated airway pressure and lung-volume effects approaching those reported during nasal HFO at similar flows [37, 38]. The downstream improvements were confined mainly to HFO-6.9 (Supplementary Material 1, Table S9), suggesting that a minimum pressure effect may be required for these physiological benefits to emerge.

### Clinical implications for ventilator liberation

Tracheal HFO during SBT may have implications for limiting excessive global lung stress. Increasing evidence suggests that the transition from supported ventilation to unsupported SBT can expose vulnerable patients to vigorous spontaneous breathing, generating large ∆P_L,dyn_ that contributes to patient self-inflicted lung injury [39, 40]. Compared with T-piece, tracheal HFO reduced ∆P_L,dyn_ by increasing the inspiratory drop in airway pressure while maintaining stable esophageal pressure swings (Supplementary Material 1, Figure S2). This finding suggests a lower dynamic mechanical load without substantial unloading of inspiratory effort.

Preservation of lung volume may also be clinically relevant. Loss of end-expiratory lung volume and increased regional ventilation inhomogeneity during T-piece trials have been associated with weaning failure and may increase stress in dependent or poorly aerated lung regions [41–43]. In this study, tracheal HFO preserved end-expiratory lung volume and improved ventilation homogeneity, which may be particularly relevant in patients prone to loss of aerated lung volume.

Tracheal HFO may also attenuate the abrupt cardiopulmonary transition created by T-piece trials. Sudden withdrawal of positive airway pressure can increase venous return and left ventricular afterload, potentially contributing to weaning-induced pulmonary edema in patients with limited cardiac reserve [44, 45]. The low-level, PEEP-like effect generated by tracheal HFO may support more gradual cardiopulmonary adaptation. At the same time, in patients at high risk of extubation failure, prophylactic noninvasive ventilation after extubation may remain the more clinically decisive intervention [46]. Thus, SBT strategy should be integrated with the post-extubation support plan in risk-tailored ventilator liberation.

An important requirement for any SBT strategy is that it should not mask clinically relevant respiratory muscle load. PSV can unload the respiratory muscles and may underestimate the burden that patients will face after extubation [5–7]. In this study, no statistically significant difference in ΔPes was observed across tracheal HFO, T-piece, and measured post-extubation values, suggesting no clear signal of substantial inspiratory-effort unloading. PTPes, WOB, and ΔPes did not differ across the five SBT phases, despite the slightly higher airway resistance with smaller expiratory ports and higher flows. This may reflect improved ventilation homogeneity and lower elastic work. However, the absence of post-extubation EIT, PTPes, and WOB measurements limited assessment of post-extubation lung volume and full inspiratory workload. In addition, inspiratory effort is a dynamic variable, and its trajectory during an SBT can provide complementary information on evolving respiratory load. Previous studies using repeated invasive and noninvasive assessment of inspiratory effort have shown that time-dependent changes in effort and respiratory mechanics during SBT may help identify patients at risk of extubation failure [47, 48]. Future studies should evaluate the relationship between tracheal HFO and more complete post-extubation physiology, including serial changes in inspiratory effort during the trial.

In relation to conventional SBT strategies, tracheal HFO creates an intermediate hybrid physiological condition between unsupported T-piece breathing and low-level positive-pressure support. It does not provide direct pressure support; instead, the interaction between continuous high flow and expiratory resistance generates a low-level PEEP-like effect. This flow-dependent support may explain the improvements in lung volume, oxygenation, and ∆P_L,dyn_, whereas the absence of direct inspiratory pressure support may explain why no statistically significant difference in ΔPes was observed across tracheal HFO, T-piece, and measured post-extubation values. Thus, tracheal HFO represents a physiologically distinct SBT strategy. Clinicians should individualize SBT selection by balancing support provision against support withdrawal according to the underlying disease and the relative concern for delayed extubation versus extubation failure. Further studies are needed to evaluate its relationship to more complete post-extubation physiology and determine whether these physiological effects translate into improved clinical outcomes.

### Limitations

Our study has several limitations. First, because some patients were unable to tolerate a tight-fitting facial mask after extubation, accurate measurements of flow, tidal volume, and derived parameters such as PTPes and WOB were not always feasible. Therefore, ΔPes was used as a surrogate marker of inspiratory effort; this variable has been shown to correlate closely with PTPes [49]. Second, many patients in our study were extubated on the day after a successful SBT. Reliable post-extubation EIT measurements for direct comparison with SBT phases were difficult to obtain because prolonged EIT monitoring after extubation is technically challenging, with possible belt displacement, changes in posture, repeated contact-gel application, and signal artifacts [43]. Future studies may consider absolute end-expiratory lung-volume measurement techniques, such as nitrogen washout/washin, although this approach also requires a sealed interface after extubation [50]. Third, a 10-min washout period was selected based on previous studies [7, 8]. In the present study, vital signs returned to baseline before each SBT phase, suggesting that the washout interval was sufficient to reduce carryover effects. Fourth, given the constraints of study duration, patient tolerance, and data quality control, a formal low-level PSV SBT condition was not included. The PSV baseline recorded before the study, delivered at PSV of 8 (6–8) cmH_2_O and PEEP of 5 (5–6) cmH_2_O, may be considered only a surrogate PSV condition. Finally, this crossover study was designed to compare short-term physiological responses within patients. Its design and sample size therefore did not permit assessment of whether different SBT strategies influence clinical outcomes, or whether obesity and disease-specific lung pathology modify the physiological effects. The cohort included no patient with body mass index >30 kg/m^2^, and subgroup sizes according to ICU admission category were small. Larger adequately powered clinical trials including more heterogeneous patient populations are needed to determine whether the observed physiological improvements translate into better patient outcomes and to identify patient phenotypes most likely to benefit from this strategy.

## Conclusion

During SBT, HFO via endotracheal tube increased airway pressure and lung volume, improved ventilation homogeneity and oxygenation. No statistically significant difference in ΔPes was observed across HFO, T-piece, and post-extubation measurements. These effects were determined by flow and expiratory port diameter, suggesting that a minimum pressure effect may be needed to produce downstream physiological benefits. Together, these findings identify tracheal HFO as a physiologically distinct SBT condition that may help guide individualized SBT selection. Whether these physiological effects translate into improved clinical outcomes requires confirmation in adequately powered clinical trials.

## Supplementary Information

Supplementary Material 1

## Data Availability

The datasets used and/or analysed during the current study are available from the corresponding author on reasonable request.

## Abbreviations

SBT: Spontaneous breathing trial
PSV: Pressure support ventilation
WOB: Work of breathing
HFO: High-flow oxygen
ICU: Intensive care unit
EIT: Electrical impedance tomography
PEEP: Positive end-expiratory pressure
HFO-9.8: HFO via endotracheal tube with a 9.8 mm expiratory port
HFO-6.9: HFO via endotracheal tube with a 6.9 mm expiratory port
EtCO_2_: End-tidal carbon dioxide pressure
V_T_: Tidal volume
∆P_L,dyn_: Dynamic transpulmonary driving pressure
∆Pes: Tidal swing of esophageal pressure during inspiration
PTPes: Esophageal pressure–time product per minute
Pmus: Pressure generated by respiratory muscle during inspiration
WOB_ela_: Elastic work of breathing
WOB_res_: Resistive work of breathing
∆EELV_glob_: Global change of end-expiratory lung volume
∆EELV_dep_: Change of end-expiratory lung volume in dependent regions
∆EELV_non__-dep_: Change of end-expiratory lung volume in nondependent regions
V_Tdep_: Tidal volume distending dependent regions
V_Tnon__-dep_: Tidal volume distending nondependent regions
COV: Center of ventilation
GI: Global inhomogeneity
HFO-8.6: HFO via endotracheal tube with an 8.6 mm expiratory port
HFO-14.6: HFO via endotracheal tube with a 14.6 mm expiratory port
CI: Confidence interval
APACHE II: Acute Physiology and Chronic Health Evaluation II
