# Supplementary material for: Physiological comparison of high-flow oxygen via endotracheal tube and T-piece strategies during spontaneous breathing trials: a randomized crossover study
Source: Crit Care. 2026 Aug 18;30:433. doi: 10.1186/s13054-026-06266-5 (PMC13488186; doi:10.1186/s13054-026-06266-5)
Supplement: Supplementary file 1 [file 13054_2026_6266_MOESM1_ESM.docx]

**Additional File 1**

**Physiological comparison of high-flow oxygen via endotracheal tube and T-piece strategies during spontaneous breathing trials: a randomized crossover study**

Shan-Shan Xu, Rui-Zhi Zhang, Jun-Tong Liu, Lin Wang, Meng-Xue Hou, Xu-An, Ming-Yue Miao, Hong-Liang Li, Yue-Fu Wang, Jian-Xin Zhou

**Methods of the clinical study**

**Patients**

Adult patients undergoing invasive mechanical ventilation for at least 24 hours ready for weaning were screened for inclusion.

Exclusion criteria included: 1) age younger than 18 years old; 2) pregnancy; 3) hemodynamic instability (mean arterial pressure < 60 mmHg, heart rate >140 or <60 beats/min); 4) respiratory and oxygenation instability (respiratory rate > 35 breaths/min or SpO_2_ <90%); 5) medical history of neuromuscular diseases or phrenic nerve injury; 6) contraindications for esophageal pressure catheter insertion, including evidence of severe coagulopathy, diagnosed or suspected esophageal varices, and history of esophageal, gastric, or lung surgery; 7) evidence of active air leak from the lung, including bronchopleural fistula, pneumothorax, pneumomediastinum, or placement of chest drainage; 8) contraindications to electrical impedance tomography (EIT) monitoring, such as the presence of a pacemaker or implantable cardioverter defibrillator; and 9) anticipating withdrawal of life support. Patients were included only once.

**Weaning process**

The readiness for weaning was evaluated by the responsible physicians following the recommended clinical guidelines [1]. All patients admitted to the participating intensive care units (ICU) were consecutively screened during the morning rounds. The criteria including: 1) improvement of the causes of intubation and mechanical ventilation; 2) no agitation or coma (Richmond Agitation Sedation Scale between -2 and +1); 3) adequate respiratory function (respiratory rate ≤ 35 breathes/min and respiratory rate / tidal volume [V_T_] < 105); 4) adequate oxygenation, SpO_2_ higher than 90% with FiO_2_ less than 0.4 and positive end–expiratory pressure (PEEP) less than 8 cm H_2_O); and 5) stable hemodynamic status (heart rate < 140 beats/min, systolic blood pressure > 90 mm Hg, no or minimal vasopressors). When all these five criteria were met, a spontaneous breathing trial (SBT) was initiated.

**Data monitoring**

Patients were positioned supine with the head of the bed elevated to 30°. An esophageal balloon catheter (Mindray catheter: LOT 22101803, Shenzhen Mindray Bio–Medical Electronics Co., Ltd., Shenzhen, China) was inserted nasally to a depth of 40–50 cm. Gastric placement of the distal tip was confirmed by aspirating gastric contents and auscultating for air insufflation. The balloon was then be inflated with 5 mL of air and connected to a pressure transducer. Proper positioning was verified by end–expiratory occlusion tests, with correct placement defined as a Δ esophageal pressure / Δ airway pressure ratio of 0.8–1.2 [2,3].

Airflow, airway pressure, and esophageal pressure tracings were collected using a heated Fleisch pneumotachograph (Vitalograph, Lenexa, Kansas) and pressure transducers (KleisTEK Engineering, Bari, Italy). The Fleisch pneumotachograph and pressure transducers were connected via rigid tubing to an ICU-Lab pressure Box (ICU Lab, KleisTEK Engineering, Bari, Italy). Signals were displayed continuously and saved on a laptop for offline analysis at a sample rate of 200 Hz (ICU–Lab 2.5 Software Package, KleisTEK Engineering, Bari, Italy).

An EIT dedicated belt containing 16 equally spaced electrodes was positioned around the patient’s thorax at the fifth or sixth intercostal space and connected to the EIT monitor (PulmoVista 500; Dräger Medical GmbH, Lübeck, Germany). A low-pass filter with 50 Hz was used to remove cardiac artifacts. The EIT images, produced through the application of small alternating electrical currents that traverse the circumference of the patient’s thorax, were continuously captured at a frequency of 20 Hz. Patient turning or movement was minimized during the trial. EIT data was analyzed offline via dedicated software (Dräger EIT Data Analysis Tool 6.3, Lübeck, Germany) [4].

**Study procedures**

In the participating ICUs, a light sedation strategy was followed according to the clinical guidelines [1,5]. Analgesia was routinely used in mechanically ventilated patients with continuous infusion of fentanyl or remifentanil. Sedation with midazolam, propofol, or dexmedetomidine was used when the patient exhibits agitation and sedation level was maintained on Richmond Agitation Sedation Scale of -2 to +1. The placement of esophageal pressure catheters was usually tolerated at this analgesia and sedation level. Otherwise, topical anesthesia to the nasal cavity and oropharynx was used.

Humidified T-piece oxygen was delivered using the air entrainment nebulizer and T-tube (Flexicare Medical Ltd, Mid Glamorgan, UK) connected to the oxygen flowmeter. High-flow oxygen (HFO) via endotracheal tube was delivered via the Aeonmed NeoHiF-i7 device (Beijing Aeonmed Company, Beijing, China) with a manufacturer’s standard assembly, including an HT-08 heated breathing circuit (Beijing Aeonmed Company, Beijing, China) and a VHC20 auto-fill humidification chamber (Inspired Medical, Guangdong, China). The tracheal HFO interface (Xiangyue Medical Ltd, Tianjin, China) was positioned between the endotracheal tube and the heated breathing circuit. The study used tracheal HFO interfaces with two different expiratory port diameters: one was 9.8 mm (LOT 20210007), and the other was 6.9 mm (LOT 20241004). Diameter was defined as the minimal internal diameter at the expiratory port of the interface (Figure S1).

In our routine weaning and extubation practice, analgesia and sedation was minimized or discontinued before SBTs [1]. Following the establishment of respiratory monitoring, a 10-minute baseline recording was obtained using the pressure support and PEEP levels previously set by the responsible physician before the patient’s inclusion. Before the start of the trial, the crossover sequence was informed by a researcher who was not involved in the trial procedures but was responsible for quality control and study supervision. The sequence was generated using a computer-based randomization process.

Then, each patient received five 15 minutes SBTs in computer-generated random order: 1) T-piece (6L/min); 2) HFO via endotracheal tube with a 9.8 mm expiratory port (HFO-9.8) at 40L/min; 3) HFO-9.8 at 60L/min; 4) HFO via endotracheal tube with a 6.9 mm expiratory port (HFO-6.9) at 40L/min; 5) HFO-6.9 at 60L/min. A 10-minute washout period with ventilator reconnection to the baseline was set between each phase.

Participants were closely monitored. Attending physicians could stop the study interventions according to the criteria for failure of SBT including: 1) respiratory rate of higher than 35 breaths/min for more than 5 min; 2) SpO_2_ of lower than 90%; 3) elevated PaCO_2_ and/or pH of less than 7.3; 4) heart rate higher than 140 beats/min or a sustained change of more than 20%; 5) systolic blood pressure greater than 180 mm Hg or less than 90 mm Hg; 6) signs of cardiac ischemia indicated by dynamic ST changes on ICU monitor or electrocardiogram; 7) abrupt unconsciousness with Richmond Agitation Sedation Scale below -3; and 8) signs of anxiety, agitation, or diaphoresis [1].

After completing all study procedures, participants were reconnected to the ventilator. Attending physicians retained the discretion to perform additional SBTs and made extubation decisions based on clinical guidelines and professional judgment [1].

Supplemental oxygen after extubation was conventionally administered via a nasal cannula at a flow of 5L/min or through other oxygen therapies, such as the nasal HFO, as determined by the clinicians’ discretion.

**Data collection**

At study entry, we collected demographic data, medical history, the diagnosis and indications for mechanical ventilation, illness severity (assessed via the Acute Physiology and Chronic Health Evaluation II [APACHE II]), duration of ventilation prior to enrollment, baseline PSV settings and ventilatory parameters, the use of analgesics and sedatives, and hemodynamic conditions.

Following the initiation of esophageal pressure and EIT monitoring, we recorded measurements of respiratory mechanics. Flow, airway pressure, esophageal pressure, and EIT data were collected during the last 3 minutes of each period, including baseline, each SBT, and washout periods. The esophageal pressure was collected for 3 minutes after extubation when the patient’s breathing condition remained stable. Ten consecutive constant breathing cycles, e.g., no coughing, no shallow breathing, no maximal inspiratory effort, no esophageal spasm, and no artifacts, were off–line assessed.

All patients were followed up until their hospital discharge, death, or 28 days post–enrollment, whichever occurred first. The following data was collected: duration of mechanical ventilation, SBT outcomes, extubation outcomes (success, reintubation, or tracheostomy), time and reasons for reintubation if extubation failure occurs, prophylactic use of noninvasive ventilation or nasal HFO, length of stay in the ICU and hospital, and all-cause mortality. Patients who were unable to tolerate the trial, had unusable data, or failed to undergo extubation were excluded to ensure the target sample size. The occurrence and underlying reasons for these exclusions were documented.

**Data measurements**

After the trial, waveform data was downloaded separately for each patient. Each segment included one SBT phase. To minimize data measurement bias, a researcher who was not involved in the measurement process assigned a three-digit code to each of the segments of waveform data from each patient. These coded segments were randomly allocated to four experienced assessors. Each assessor evaluated waveform segments derived from various experimental conditions in different patients.

1. Airway pressure and flow derived parameters

1) The mean and peak inspiratory and expiratory airway pressure were measured with the points of zero flow to identify the inspiration and expiration phases. PEEP was measured at the end of the expiratory phase with zero flow [6].

2) The mean and peak inspiratory and expiratory flow rate were measured with the points of zero flow to identify inspiration and expiration phases.

3) The inspiratory tidal volume (V_T_) was integrated from the flow tracing. Respiratory rate was counted as the breaths at the last minute. The minute ventilation was calculated from respiratory rate and V_T_. The inspiratory (Ti) and expiratory (Te) times, along with the inspiratory to expiratory (I:E) ratio, were also measured.

4) FiO_2_ during T-piece was estimated as:

FiO_2_=[(Ti×100×1.0)+(V_T_–Ti×100×1.0)×0.21)]/V_T_

Ti was inspiratory time (s), 100 represented T–piece oxygen flow rate (6 L/min = 100 mL/s), and 0.21 represented oxygen concentration in room air [7].

2. Esophageal pressure derived parameters [4,8]

1) The tidal swing of esophageal pressure during inspiration (ΔPes) was measured as the difference between the minimum value throughout the respiratory cycle and the value at the onset of the inspiratory effort. The onset of inspiratory effort was defined as the point of negative deflection of esophageal pressure with a rapid change in slope.

2) The esophageal pressure–time product per breath (PTP per breath) was derived as the area between the esophageal pressure and the estimated recoil pressure of the chest wall from the onset of inspiratory effort to the end of inspiration (zero flow). The esophageal pressure–time product per minute (PTPes) was derived by multiplying the PTP per breath by respiratory rate. The chest wall compliance was approximated as 4% of the predicted vital capacity calculated based on patients’ age and height [9]. The estimated chest wall recoil pressure was calculated as volume curve divided by chest wall compliance.

3) The respiratory muscle pressure during inspiration (Pmus) was calculated as the maximal difference between the esophageal pressure and the estimated recoil pressure of the chest wall.

4) The work of breathing (WOB) per breath was calculated from the Campbell diagram by integrating the area between the esophageal pressure/V_T_ curve and the static esophageal pressure/V_T_ curve of the chest wall. Inspiratory WOB was expressed as the work per time unit (joules per minute).

5) The transpulmonary pressure (P_L_) was measured as the difference between airway pressure and esophageal pressure. The dynamic transpulmonary driving pressure (∆P_L,dyn_) was calculated as the difference between the maximum value of P_L_ and the value at the onset of the inspiratory effort [10].

6) The inspiratory and expiratory airway resistance were estimated at the flow rate of 0.2 L/s using the method introduced by Mead et al.:

R=[(Pes0–Pes)–(V/C)]/V’

Where R was the resistance, Pes0 was the esophageal pressure at the start of inspiratory or expiratory flow, V was the instantaneous volume integrated from flow, C was the dynamic compliance obtained for the same breath as the ratio of Vt to ∆Pes, and V’ was the instantaneous flow rate (0.2 L/s) [11].

3. EIT data analysis

During the PSV baseline, an end-expiratory occlusion was performed to generate synchronous changes in airway pressure, flow, and EIT tracings. We selected the same 20 breaths preceding the simultaneous change from the flow and EIT tracings. The ratio between milliliters and arbitrary units of tidal impedance variations was calculated, allowing conversion of impedance changes into lung volume changes throughout all study phase [12,13]. We divided the EIT lung-imaging field from ventral to dorsal into four regions of interest (ROI). ROI1 and ROI2 were combined to represent the non-dependent region, while ROI3 and ROI4 constituted the dependent region.

1) The dependent fraction of ventilation: the percentage of ventilation reaching the dependent region (ROI3 and ROI4).

2) The dependent and non–dependent V_T_ (V_Tdep_ and V_Tnon-dep_) were derived by multiplying the average global V_T_ (V_Tglob_) by the respective proportion of regional ventilation.

3) Global and regional changes in end-expiratory lung volume (∆EELV) during each SBT phase were calculated relative to the PSV baseline recorded before the five randomized SBT phases. ∆EELV was calculated as the change in end-expiratory impedance from this baseline multiplied by the individual volume-impedance calibration ratio.

4) The center of ventilation (COV) was calculated as the percentage of tidal ventilation distributed to the dorsal region of the global lung. A higher COV signifies a greater distribution of tidal ventilation to the dependent region [14].

5) The global inhomogeneity (GI) index was calculated as a measure of inhomogeneous distribution of tidal ventilation [15].

**Methods of the bench experiment**

**Experimental setup**

A size 8.0 endotracheal tube (Shiley, Covidien, Mansfield, MA) was inserted through the mouth to the trachea of the RespiPatient manikin (IngMar Medical, USA). To prevent any system leakage, the endotracheal tube cuff was inflated to its maximum capacity. The distal end of the trachea was connected to an active lung simulator (ASL 5000, IngMar Medical, USA) via a standard rigid breathing circuit to mimic patient respiratory mechanics.

**Model simulation**

Specific pulmonary mechanics and effort patterns were programmed using the ASL 5000 software (version 3.6). A sinusoidal half-wave inspiratory effort was simulated with rise time of 20%, inspiratory holding time of 0% and releasing time of 20%. To replicate representative mechanically ventilated patients during SBT, 24 spontaneous breathing models were simulated:

- Respiratory system compliance: 40 and 60 mL/cmH_2_O
- Airway resistance: 10 and 20 cmH_2_O s/L
- Pmus: -5, -10, and -15 cmH_2_O
- Respiratory rate: 20 and 30 breaths/min.

Values for respiratory system compliance and airway resistance were adapted from Arnal et al., representing three distinct clinical phenotypes: Normal (normal mechanics), ARDS-like (low compliance), and COPD-like (high resistance) [16]. The ranges for Pmus and respiratory rate were selected based on previously reported bench models and real-world clinical data of patients undergoing SBT [17,18]. By combining these parameters, our model systematically reflects the broad spectrum of respiratory drive and mechanics encountered in the ICU, ensuring the generalizability of the interface performance evaluation.

**Tracheal HFO interfaces**

Four tracheal HFO interfaces with different expiratory port diameters were evaluated (Figure S3):

- HFO-6.9: Xiangyue Medical Ltd, Tianjin, China
- HFO via endotracheal tube with a 8.6 mm expiratory port (HFO-8.6): Veoflo, Flexicare, Mountain Ash, United Kingdom
- HFO-9.8: Xiangyue Medical Ltd, Tianjin, China
- HFO via endotracheal tube with a 14.6 mm expiratory port (HFO-14.6): OPT970 Optiflow+, Fisher & Paykel, Auckland, New Zealand

For each interface, six flow settings were evaluated (10, 20, 30, 40, 50, and 60 L/min) with the HFT FiO_2_ setting at 0.21 and temperature of 37°C.

**Data analysis**

The flow, airway pressure and Pmus was recorded for 5 min at each tracheal HFO flow rate through ASL software 3.6 at 512 Hz. Last 10 breaths of each record were used for the off-line data analysis.

The primary outcomes of bench study were the mean expiratory airway pressure and PEEP. The second outcomes included the following physiological parameters: ∆EELV, ∆P_L,dyn_, VT, peak flow, and airway resistance.

Bench data analysis was conducted according to the same methodology described for the human study. ΔEELV in bench study was estimated using a single-breath disconnection maneuver adapted from a previous study [19]. After a steady-state period under each experimental condition, respiratory rate was transiently reduced to 10 breaths per minute to facilitate complete expiration. The circuit was then abruptly disconnected, and the difference in expiratory tidal volume between the last breath before disconnection and the first breath after disconnection was used to estimate ΔEELV. This approach reflects the change in volume associated with removal of flow-generated positive airway pressure, as the model does not reproduce alveolar recruitment.

**Statistical analysis**

Continuous variables are presented as median with interquartile range, and categorical variables are reported as counts and percentages. Given the small sample size, comparisons across the five SBT conditions and the post–extubation phase were performed using the Friedman test, with Dunn’s test and Bonferroni correction for post hoc pairwise comparisons. As each participant underwent all study phases and randomization was expected to balance potential sequence–related effects, no adjustment for trial order was applied.

For sample size calculation of this human physiological study, we adopted a Cohen–based medium effect size of 0.25 alongside a statistical power of 0.8 and a significance level set at 0.05 to achieve adequate statistical power [20]. The correlation among repeated measures was specified as 0.5, and the nonsphericity correction (ε) was set to 1.0, in accordance with conservative default assumptions. Consequently, the sample size calculation yielded a requirement of 20 subjects using the GPower version 3.1.9.7. Patients who were unable to complete all study phases due to SBT intolerance, unsuccessful extubation, or missing post–extubation esophageal pressure measurements would be excluded. Additional patients were recruited to guarantee that final analysis included 20 subjects with complete SBTs and post–extubation datasets.

In the bench study, continuous variables are reported as mean ± SD in descriptive tables. A linear mixed–effects model was adopted to evaluate the performance of different tracheal HFO interfaces across a broad range of clinical conditions. We simulated 24 unique respiratory scenarios to represent a wide spectrum of clinical phenotypes. Expiratory port diameter, flow rate, and their interaction were defined as fixed effects. The 24 scenarios were treated as a random effect to account for the baseline variability in different simulated phenotypes, allowing for a generalized assessment of interface performance. Model-estimated marginal means with 95% confidence intervals were derived for graphical presentation.

Statistical analysis was performed using SPSS IBM Version 26.0, GraphPad Prism 9.0, and R (version 4.5.2) software. A p-value of less than 0.05 is considered statistically significant.

**References**

1. Boles JM, Bion J, Connors A, Herridge M, Marsh B, Melot C, et al. Weaning from mechanical ventilation. Eur Respir J. 2007;29:1033-56.

2. Baydur A, Behrakis PK, Zin WA, Jaeger M, Milic-Emili J. A simple method for assessing the validity of the esophageal balloon technique. Am Rev Respir Dis. 1982;126:788-91.

3. Higgs BD, Behrakis PK, Bevan DR, Bevan JC, Milic-Emili J. Measurement of pleural pressure with esophageal balloon in anesthetized humans. Anesthesiology. 1983;59:340-3.

4. Mauri T, Yoshida T, Bellani G, Goligher EC, Carteaux G, Rittayamai N, et al. Esophageal and transpulmonary pressure in the clinical setting: meaning, usefulness and perspectives. Intensive Care Med. 2016;42:1360-73.

5. Devlin JW, Skrobik Y, Gelinas C, Needham DM, Slooter AJC, Pandharipande PP, et al. Clinical practice guidelines for the prevention and management of pain, agitation/sedation, delirium, immobility, and sleep disruption in adult patients in the ICU. Crit Care Med. 2018;46:e825-e873.

6. Chen GQ, Sun XM, Wang YM, He X, Hu SL, Li HL, et al. Additional expiratory resistance elevates airway pressure and lung volume during high-flow tracheal oxygen via tracheostomy. Sci Rep. 2019;9:14542.

7. Kacmarek RM, Stoller JK, Heuer AJ. Egan's fundamentals of respiratory care. 11th ed. St. Louis, MO: Elsevier Health Sciences Division; 2016.

8. Akoumianaki E, Maggiore SM, Valenza F, Bellani G, Jubran A, Loring SH, et al. The application of esophageal pressure measurement in patients with respiratory failure. Am J Respir Crit Care Med. 2014;189:520-31.

9. Jaber S, Sebbane M, Verzilli D, Matecki S, Wysocki M, Eledjam JJ, et al. Adaptive support and pressure support ventilation behavior in response to increased ventilatory demand. Anesthesiology. 2009;110:620-7.

10. Bertoni M, Telias I, Urner M, Long M, Del Sorbo L, Fan E, et al. A novel non-invasive method to detect excessively high respiratory effort and dynamic transpulmonary driving pressure during mechanical ventilation. Crit Care. 2019;23:346.

11. Mead J, Whittenberger JL. Physical properties of human lungs measured during spontaneous respiration. J Appl Physiol. 1953;5:779-96.

12. Mauri T, Eronia N, Turrini C, Battaglini D, Grasselli G, Jak S, et al. Bedside assessment of the effects of positive end-expiratory pressure on lung inflation and recruitment by the helium dilution technique and electrical impedance tomography. Intensive Care Med. 2016;42:1576-87.

13. Mauri T, Alban L, Turrini C, Cambiaghi B, Carlesso E, Taccone P, et al. Optimum support by high-flow nasal cannula in acute hypoxemic respiratory failure: effects of increasing flow rates. Intensive Care Med. 2017;43:1453-63.

14. Frerichs I, Dargaville PA, van Genderingen H, Morel DR, Rimensberger PC. Lung volume recruitment after surfactant administration modifies spatial distribution of ventilation. Am J Respir Crit Care Med. 2006;174:772-9.

15. Zhao Z, Pulletz S, Frerichs I, Müller-Lisse U, Möller K. The EIT-based global inhomogeneity index is highly correlated with regional lung opening in patients with acute respiratory distress syndrome. BMC Res Notes. 2014;7:82.

16. Arnal JM, Garnero A, Saoli M, Chatburn RL. Parameters for simulation of adult subjects during mechanical ventilation. Respir Care. 2018;63:158-68.

17. Rigault G, Guérin C, Sigaud F, Argaud L, Galerneau LM, Terzi N. Bench study of a spontaneous breathing trial with different modalities. Respir Care. 2023;68:760-6.

18. Capdevila M, Aarab Y, Monet C, De Jong A, Vonarb A, Carr J, et al. Spontaneous breathing trials should be adapted for each patient according to the critical illness: the GLOBAL WEAN study. Intensive Care Med. 2024;50:2083-93.

19. Chen L, Chen GQ, Shore K, Shklarren M, Martins C, Winkler MH, et al. Implementing a bedside assessment of respiratory mechanics in patients with acute respiratory distress syndrome. Crit Care. 2017;21:84.

20. Cohen J. Statistical power analysis for the behavioral sciences. 2nd ed. Hillsdale, NJ: Lawrence Erlbaum Associates; 1988.

**Tables**

**Table S1.** Randomization Table

| Patient No. | SBT Phase 1 | SBT Phase 2 | SBT Phase 3 | SBT Phase 4 | SBT Phase 5 |
| --- | --- | --- | --- | --- | --- |
| 1 | T–piece | HFO−6.9 (60 L/min) | HFO−9.8 (40 L/min) | HFO−9.8 (60 L/min) | HFO−6.9 (40 L/min) |
| 2 | HFO−6.9 (60 L/min) | HFO−9.8 (60 L/min) | HFO−9.8 (40 L/min) | T–piece | HFO−6.9 (40 L/min) |
| 3 | T–piece | HFO−9.8 (40 L/min) | HFO−9.8 (60 L/min) | HFO−6.9 (60 L/min) | HFO−6.9 (40 L/min) |
| 4 | HFO−9.8 (40 L/min) | T–piece | HFO−6.9 (40 L/min) | HFO−9.8 (60 L/min) | HFO−6.9 (60 L/min) |
| 5 | HFO−9.8 (60 L/min) | HFO−9.8 (40 L/min) | HFO−6.9 (60 L/min) | T–piece | HFO−6.9 (40 L/min) |
| 6 | HFO−6.9 (60 L/min) | HFO−9.8 (40 L/min) | T–piece | HFO−9.8 (60 L/min) | HFO−6.9 (40 L/min) |
| 7 | HFO−9.8 (40 L/min) | T–piece | HFO−6.9 (40 L/min) | HFO−6.9 (60 L/min) | HFO−9.8 (60 L/min) |
| 8 | HFO−6.9 (60 L/min) | HFO−6.9 (40 L/min) | HFO−9.8 (60 L/min) | HFO−9.8 (40 L/min) | T–piece |
| 9 | T–piece | HFO−9.8 (40 L/min) | HFO−6.9 (40 L/min) | HFO−9.8 (60 L/min) | HFO−6.9 (60 L/min) |
| 10 | HFO−6.9 (60 L/min) | HFO−9.8 (40 L/min) | HFO−6.9 (40 L/min) | T–piece | HFO−9.8 (60 L/min) |
| 11 | HFO−6.9 (40 L/min) | HFO−9.8 (40 L/min) | HFO−6.9 (60 L/min) | T–piece | HFO−9.8 (60 L/min) |
| 12 | T–piece | HFO−6.9 (40 L/min) | HFO−6.9 (60 L/min) | HFO−9.8 (60 L/min) | HFO−9.8 (40 L/min) |
| 13 | HFO−6.9 (60 L/min) | HFO−9.8 (40 L/min) | HFO−6.9 (40 L/min) | T–piece | HFO−9.8 (60 L/min) |
| 14 | HFO−9.8 (40 L/min) | HFO−9.8 (60 L/min) | HFO−6.9 (40 L/min) | HFO−6.9 (60 L/min) | T–piece |
| 15 | HFO−6.9 (60 L/min) | HFO−6.9 (40 L/min) | T–piece | HFO−9.8 (60 L/min) | HFO−9.8 (40 L/min) |
| 16 | HFO−9.8 (60 L/min) | HFO−6.9 (40 L/min) | HFO−9.8 (40 L/min) | HFO−6.9 (60 L/min) | T–piece |
| 17 | HFO−9.8 (60 L/min) | HFO−6.9 (40 L/min) | HFO−9.8 (40 L/min) | T–piece | HFO−6.9 (60 L/min) |
| 18 | T–piece | HFO−9.8 (40 L/min) | HFO−9.8 (60 L/min) | HFO−6.9 (40 L/min) | HFO−6.9 (60 L/min) |
| 19 | HFO−9.8 (60 L/min) | HFO−6.9 (60 L/min) | T–piece | HFO−9.8 (40 L/min) | HFO−6.9 (40 L/min) |
| 20 | T–piece | HFO−6.9 (60 L/min) | HFO−9.8 (60 L/min) | HFO−9.8 (40 L/min) | HFO−6.9 (40 L/min) |

SBT: spontaneous breathing trial; HFO-9.8: HFO via endotracheal tube with a 9.8 mm expiratory port; HFO-6.9: HFO via endotracheal tube with a 6.9 mm expiratory port.

**Table S2.** Detailed airway pressure, lung volume, and gas exchange with complete pairwise comparisons across the five study phases

|  | T–piece | HFO−9.8  (40L/min) | HFO−9.8  (60L/min) | HFO−6.9  (40L/min) | HFO−6.9  (60L/min) | *p*-value |
| --- | --- | --- | --- | --- | --- | --- |
| Airway pressure | | | | | | |
| PEEP, cm H_2_O | 0.0 (0.0–0.1) | 0.9 (0.8–1.0) ^*^ | 1.9 (1.9–2.1) ^*†^ | 2.8 (2.7–3.0) ^*†‡^ | 6.0 (5.8–6.2) ^*†‡§^ | ＜0.001 |
| Peak inspiratory airway pressure, cm H₂O | −0.3 (−0.5–−0.2) | −0.1 (−0.3–0.1) ^*^ | 0.5 (0.2–0.8) ^*†^ | 0.3 (0.0–0.7) ^*†‡^ | 2.5 (1.8–2.8) ^*†‡§^ | ＜0.001 |
| Mean inspiratory airway pressure, cm H₂O | −0.1 (−0.3–−0.1) | 0.2 (0.1–0.4) ^*^ | 1.0 (0.8–1.2) ^*†^ | 1.0 (0.8–1.3) ^*†^ | 3.5 (3.0–3.6) ^*†‡§^ | ＜0.001 |
| Peak expiratory airway pressure, cm H₂O | 0.3 (0.2–0.3) | 1.9 (1.8–2.1) ^*^ | 3.2 (3.0–3.6) ^*†^ | 4.4 (4.1–4.7) ^*†‡^ | 8.0 (7.5–8.1) ^*†‡§^ | ＜0.001 |
| Mean expiratory airway pressure, cm H₂O | 0.1 (0.1–0.2) | 1.4 (1.2–1.5) ^*^ | 2.6 (2.4–2.8) ^*†^ | 3.7 (3.4–4.0) ^*†‡^ | 7.2 (6.8–7.6) ^*†‡§^ | ＜0.001 |
| Lung volume and homogeneity | | | | | | |
| Vt_non–dep_, mL | 205 (183–279) | 221 (181–305) | 206 (172–304) | 209 (156–254) ^†^ | 205 (154–254) ^†^ | 0.004 |
| Vt_dep_, mL | 186 (138–289) | 183 (134–257) | 191 (126–281) | 192 (126–265) | 216 (131–284) | 0.35 |
| ∆EELV_glob_, mL | −532 (−673–−431) | −402 (−539–−367) ^*^ | −345 (−426–−295) ^*^ | −296 (−348–−249) ^*†‡^ | −164 (−199–−106) ^*†‡§^ | < 0.001 |
| ∆EELV_dep_, mL | −224 (−390–−185) | −197 (−314–−140) ^*^ | −163 (−243–−128) ^*^ | −155 (−189–−97) ^*†‡^ | −62 (−102–−43) ^*†‡§^ | < 0.001 |
| ∆EELV_non–dep_, mL | −285 (−334–−212) | −231 (−265–−182) ^*^ | −185 (−228–−144) ^*^ | −146 (−189–−101) ^*†‡^ | −79 (−109–−42) ^*†‡§^ | < 0.001 |
| Dependent fraction of ventilation, % | 46 (38–55) | 46 (34–56) | 46 (33–58) | 53 (37–60) ^*^ | 53 (35–60) ^*†‡^ | 0.001 |
| COV, % | 48 (44–51) | 47 (43–51) | 48 (44–53) | 48 (44–51) | 48 (44–53) | 0.17 |
| GI index | 0.53 (0.49–0.58) | 0.51 (0.49–0.58) | 0.51 (0.46–0.58) | 0.51 (0.43–0.54) | 0.49 (0.43–0.52) ^*‡^ | 0.003 |
| Gas exchange |  |  |  |  |  |  |
| pH | 7.43 (7.42–7.44) | – | 7.42 (7.41–7.45) | – | 7.42 (7.40–7.45) | 0.14 |
| PaO_2_, mm Hg | 105 (86–121) | – | 109 (92–120) | – | 121 (102–142) ^*‡^ | 0.001 |
| PaCO_2_, mm Hg | 41 (38–43) | – | 40 (38–44) | – | 41 (38–45) | 0.11 |
| PaO_2_/FiO_2_, mm Hg | 268 (219–316) | – | 273 (229–299) | – | 303 (256–354) ^*‡^ | ＜0.001 |

HFO-9.8: HFO via endotracheal tube with a 9.8 mm expiratory port; HFO-6.9: HFO via endotracheal tube with a 6.9 mm expiratory port; PEEP: positive end-expiratory pressure; VT_dep_: tidal volume distending dependent regions; VT_non-dep_: tidal volume distending nondependent regions; ∆EELV_glob_: global change of end-expiratory lung volume; ∆EELV_dep_: change of end-expiratory lung volume in dependent regions; ∆EELV_non–dep_: change of end-expiratory lung volume in nondependent regions; COV: center of ventilation; GI index: global inhomogeneity index*.*

Data are presented as median (interquartile range). *p*-values represent global differences across the five conditions determined by the Friedman test. Post hoc pairwise comparisons were performed using Dunn’s test with Bonferroni correction.

^*^*p* < 0.05 versus T–piece.

^†^*p* < 0.05 versus HFO–9.8 (40L/min).

^‡^*p* < 0.05 versus HFO–9.8 (60L/min).

^§^*p* < 0.05 versus HFO–6.9 (40L/min).

**Table S3.** Detailed hemodynamics, respiratory variables, and inspiratory effort with complete pairwise comparisons across the five study phases

|  | T–piece | HFO−9.8  (40L/min) | HFO−9.8  (60L/min) | HFO−6.9  (40L/min) | HFO−6.9  (60L/min) | *p-*value |
| --- | --- | --- | --- | --- | --- | --- |
| Hemodynamics | | | | | | |
| Heart rate, bpm | 89 (79–101) | 90 (75–104) | 90 (78–103) | 92 (79–105) | 91 (77–103) | 0.80 |
| Mean blood pressure, mm Hg | 95 (85–104) | 92 (85–105) | 94 (87–104) | 93 (86–105) | 95 (88–106) | 0.42 |
| SpO_2_, % | 99 (97–100) | 99 (98–100) | 99 (98–100) | 99 (98–100) | 99 (98–100) | 0.36 |
| Respiratory variables | | | | | | |
| Respiratory rate, breaths/min | 22 (16–27) | 20 (16–27) | 20 (16–27) | 20 (16–25) ^*^ | 19 (16–24) ^*^ | 0.002 |
| V_T_, mL | 421 (339–530) | 438 (353–558) | 423 (331–553) | 423 (321–530) | 429 (342–523) | 0.22 |
| Minute ventilation, L/min | 9.0 (7.2–10.4) | 8.7 (6.6–9.9) | 8.8 (6.3–9.7) | 8.5 (6.4–9.3) ^*†^ | 8.3 (6.1–9.5) ^*†‡^ | ＜0.001 |
| EtCO_2_, mm Hg | 39 (32–43) | 39 (32–43) | 39 (32–43) | 39 (32–43) | 39 (32–43) | 0.11 |
| Expiratory time, s | 1.67 (1.44–2.78) | 1.99 (1.46–2.60) | 1.85 (1.43–2.63) | 1.74 (1.53–2.72) | 2.00 (1.64–2.47) | 0.10 |
| Expiratory time/ total respiratory time, % | 64 (62–70) | 65 (62–71) | 65 (62–71) | 65 (63–70) | 67 (62–70) | 0.11 |
| Peak inspiratory flow, L/s | 0.63 (0.49–0.80) | 0.62 (0.50–0.73) | 0.61 (0.48–0.78) | 0.60 (0.45–0.71) | 0.57 (0.46–0.70) ^*^ | 0.005 |
| Peak expiratory flow, L/s | 0.42 (0.38–0.50) | 0.41 (0.35–0.45) | 0.40 (0.32–0.43) ^*^ | 0.35 (0.31–0.42) ^*†‡^ | 0.32 (0.27–0.37) ^*†‡§^ | ＜0.001 |
| Inspiratory airway resistance, cm H₂O s/L | 5.1 (3.8–7.3) | 6.5 (4.4–9.7) | 7.1 (4.5–10.1) ^*^ | 7.4 (5.4–11.6) ^*‡^ | 7.9 (5.4–12.8) ^*†‡^ | ＜0.001 |
| Expiratory airway resistance, cm H₂O s/L | 6.5 (4.2–8.8) | 7.8 (6.3–8.9) ^*^ | 7.9 (6.4–9.7) ^*^ | 9.6 (6.9–11.8) ^*†^ | 10.3 (8.4–13.9) ^*†‡§^ | ＜0.001 |
| Inspiratory effort |  |  |  |  |  |  |
| ∆Pes, cm H_2_O | 15 (11–16) | 14 (10–17) | 13 (10–17) | 14 (10–17) | 13 (10–16) | 0.99 |
| PTPes, cm H₂O s/min | 207 (157–251) | 207 (158–234) | 212 (151–242) | 211 (155–247) | 214 (156–260) | 0.31 |
| Pmus, cm H_2_O | 17 (14–20) | 17 (13–21) | 16 (13–20) | 16 (13–21) | 15 (12–21) | 0.84 |
| ∆P_L,dyn_, cm H_2_O | 14 (10–16) | 13 (9–16) | 12 (9–15) ^*^ | 11 (8–14) ^*†‡^ | 9 (7–13) ^*†‡§^ | ＜0.001 |
| WOB, J/min | 8.7 (7.4–11.5) | 8.9 (7.2–11.0) | 8.4 (6.8–12.1) | 9.3 (7.2–10.7) | 9.3 (6.7–10.4) | 0.86 |
| WOB_res_, J/min | 3.9 (2.4–5.5) | 3.8 (2.9–4.9) | 3.9 (3.0–5.8) | 4.2 (3.1–5.3) | 4.1 (3.2–5.5) | 0.40 |
| WOB_ela_, J/min | 5.1 (3.9–6.5) | 4.9 (4.2–5.9) | 5.0 (3.7–5.9) | 4.9 (3.9–5.6) ^*†^ | 4.8 (3.7–5.4) ^*†^ | 0.007 |

HFO-9.8: HFO via endotracheal tube with a 9.8 mm expiratory port; HFO-6.9: HFO via endotracheal tube with a 6.9 mm expiratory port; V_T_: tidal volume; EtCO_2_: end-tidal carbon dioxide pressure; ∆Pes: tidal swing of esophageal pressure during inspiration; PTPes: esophageal pressure time product per minute; Pmus: pressure generated by respiratory muscle during inspiration; ∆P_L,dyn_: dynamic transpulmonary driving pressure; WOB: work of breathing; WOB_res_: resistive work of breathing; WOB_ela_: elastic work of breathing.

Data are presented as median (interquartile range). *p*-values represent global differences across the five conditions determined by the Friedman test. Post hoc pairwise comparisons were performed using Dunn’s test with Bonferroni correction.

^*^*p* < 0.05 versus T–piece.

^†^*p* < 0.05 versus HFO–9.8 (40L/min).

^‡^*p* < 0.05 versus HFO–9.8 (60L/min).

^§^*p* < 0.05 versus HFO–6.9 (40L/min).

**Table S4.** Physiological variables at PSV baseline and across the five study phases

|  | PSV Baseline  PEEP 5 (5-6) cmH_2_O,  PSV 8 (6-8) cmH_2_O | T–piece | HFO−9.8  (40L/min) | HFO−9.8  (60L/min) | HFO−6.9  (40L/min) | HFO−6.9  (60L/min) | *p-*value |
| --- | --- | --- | --- | --- | --- | --- | --- |
| Respiratory rate, breaths/min | 17 (14–23) | 22 (16–27) ^*^ | 20 (16–27) ^*^ | 20 (16–27) ^*^ | 20 (16–25) ^*†^ | 19 (16–24) ^*†^ | ＜0.001 |
| V_T_, mL | 508 (417–646) | 421 (339–530) ^*^ | 438 (353–558) ^*^ | 423 (331–553) ^*^ | 423 (321–530) ^*^ | 429 (342–523) ^*^ | ＜0.001 |
| Minute ventilation, L/min | 9.7 (6.9–10.5) | 9.0 (7.2–10.4) | 8.7 (6.6–9.9) | 8.8 (6.3–9.7) | 8.5 (6.4–9.3) ^*^ | 8.3 (6.1–9.5) ^*^ | 0.014 |
| Lung volume and homogeneity | | | | | | | |
| Vt_non–dep_, mL | 232 (188–321) | 205 (183–279) | 221 (181–305) | 206 (172–304) | 209 (156–254) ^*‡^ | 205 (154–254) ^*‡^ | 0.001 |
| Vt_dep_, mL | 263 (172–349) | 186 (138–289) ^*^ | 183 (134–257) ^*^ | 191 (126–281) ^*^ | 192 (126–265) ^*^ | 216 (131–284) ^*^ | ＜0.001 |
| ∆EELV_glob_, mL | Reference | −532 (−673–−431) | −402 (−539–−367) ^*†^ | −345 (−426–−295) ^*†‡^ | −296 (−348–−249) ^*†‡§^ | −164 (−199–−106) ^*†‡§ll^ | ＜0.001 |
| ∆EELV_dep_, mL | Reference | −224 (−390–−185) | −197 (−314–−140) ^*†^ | −163 (−243–−128) ^*†‡^ | −155 (−189–−97) ^*†‡§^ | −62 (−102–−43) ^*†‡§ll^ | ＜0.001 |
| ∆EELV_non–dep_, mL | Reference | −285 (−334–−212) | −231 (−265–−182) ^*†^ | −185 (−228–−144) ^*†‡^ | −146 (−189–−101) ^*†‡§^ | −79 (−109–−42) ^*†‡§ll^ | ＜0.001 |
| Dependent fraction of ventilation, % | 55 (36–62) | 46 (38–55) ^*^ | 46 (34–56) ^*^ | 46 (33–58) ^*^ | 53 (37–60) ^*^ | 53 (35–60) | 0.002 |
| COV, % | 49 (44–53) | 48 (44–51) | 47 (43–51) | 48 (44–53) | 48 (44–51) | 48 (44–53) | 0.06 |
| GI index | 0.47 (0.41–0.50) | 0.53 (0.49–0.58) ^*^ | 0.51 (0.49–0.58) ^*^ | 0.51 (0.46–0.58) ^*^ | 0.51 (0.43–0.54) ^*^ | 0.49 (0.43–0.52) ^†‡§^ | ＜0.001 |
| Inspiratory effort | | | | | | | |
| ∆Pes, cm H_2_O | 8 (7–14) | 15 (11–16) ^*^ | 14 (10–17) ^*^ | 13 (10–17) ^*^ | 14 (10–17) ^*^ | 13 (10–16) ^*^ | ＜0.001 |
| PTPes, cm H₂O s/min | 143 (128–209) | 207 (157–251) ^*^ | 207 (158–234) ^*^ | 212 (151–242) ^*^ | 211 (155–247) ^*^ | 214 (156–260) ^*^ | 0.008 |
| Pmus, cm H_2_O | 12 (10–18) | 17 (14–20) ^*^ | 17 (13–21) ^*^ | 16 (13–20) ^*^ | 16 (13–21) ^*^ | 15 (12–21) ^*^ | ＜0.001 |
| ∆P_L,dyn_, cm H_2_O | 14 (11–18) | 14 (10–16) | 13 (9–16) | 12 (9–15) ^*^ | 11 (8–14) ^*†‡§^ | 9 (7–13) ^*†‡§ll^ | ＜0.001 |
| WOB, J/min | 7.1 (5.0–13.4) | 8.7 (7.4–11.5) | 8.9 (7.2–11.0) | 8.4 (6.8–12.1) | 9.3 (7.2–10.7) | 9.3 (6.7–10.4) | 0.177 |
| WOB_res_, J/min | 2.3 (1.5–5.6) | 3.9 (2.4–5.5) | 3.8 (2.9–4.9) | 3.9 (3.0–5.8) | 4.2 (3.1–5.3) | 4.1 (3.2–5.5) | 0.075 |
| WOB_ela_, J/min | 3.0 (2.2–4.7) | 5.1 (3.9–6.5) | 4.9 (4.2–5.9) | 5.0 (3.7–5.9) | 4.9 (3.9–5.6) | 4.8 (3.7–5.4) | 0.003 |

PSV: pressure support ventilation; PEEP: positive end-expiratory pressure; HFO-9.8: HFO via endotracheal tube with a 9.8 mm expiratory port; HFO-6.9: HFO via endotracheal tube with a 6.9 mm expiratory port; V_T_: tidal volume; V_Tdep_: tidal volume distending dependent regions; V_Tnon–dep_: tidal volume distending nondependent regions; ∆EELV_glob_: global change of end–expiratory lung volume; ∆EELV_dep_: change of end–expiratory lung volume in dependent regions; ∆EELV_non–dep_: change of end–expiratory lung volume in nondependent regions; COV: center of ventilation; GI index: global inhomogeneity index; ∆Pes: tidal swing of esophageal pressure during inspiration; PTPes: esophageal pressure time product per minute; Pmus: pressure generated by respiratory muscle during inspiration; ∆P_L,dyn_: dynamic transpulmonary driving pressure; WOB: work of breathing; WOB_res_: resistive work of breathing; WOB_ela_: elastic work of breathing.

Data are presented as median (interquartile range). *p*-values represent global differences across the five study phases and PSV baseline determined by the Friedman test. Post hoc pairwise comparisons were performed using Dunn’s test with Bonferroni correction.

**p* < 0.05 versus PSV Baseline.

^†^*p* < 0.05 versus T-piece.

^‡^*p* < 0.05 versus HFO–9.8 (40L/min).

^§^*p* < 0.05 versus HFO–9.8 (60L/min).

^ll^*p* < 0.05 versus HFO–6.9 (40L/min).

|  | PSV baseline  PEEP 5 (5-6) cmH_2_O,  PSV 8 (6-8) cmH_2_O | T–piece | HFO−9.8  (40L/min) | HFO−9.8  (60L/min) | HFO−6.9  (40L/min) | HFO−6.9  (60L/min) | Post–extubation | *p*-value |
| --- | --- | --- | --- | --- | --- | --- | --- | --- |
| Respiratory rate, breaths/min | 17 (14–23) | 22 (16–27) ^*^ | 20 (16–27) ^*^ | 20 (16–27) ^*^ | 20 (16–25) ^*^ | 19 (16–24) | 19 (16–25) | ＜0.001 |
| Heart rate, beats/min | 89 (77-100) | 89 (79–101) | 90 (75–104) | 90 (78–103) | 92 (79–105) | 91 (77–103) | 90 (80–100) | 0.78 |
| Mean blood pressure, mm Hg | 93 (85-104) | 95 (85–104) | 92 (85–105) | 94 (87–104) | 93 (86–105) | 95 (88–106) | 95 (89–104) | 0.43 |
| SpO_2_, % | 100 (98-100) | 99 (97–100) | 99 (98–100) | 99 (98–100) | 99 (98–100) | 99 (98–100) | 99 (98–99) | 0.19 |
| ∆Pes, cm H_2_O | 8 (7–14) | 15 (11–16) ^*^ | 14 (10–17) ^*^ | 13 (10–17) ^*^ | 14 (10–17) ^*^ | 13 (10–16) ^*^ | 15 (11–17) ^*^ | ＜0.001 |

**Table S5.** Physiological and esophageal pressure variables at PSV baseline, across the five study phases, and after extubation

Paired differences in ∆Pes versus post-extubation measurements

| Comparison | Median paired difference, cm H_2_O | 95% bootstrap CI |
| --- | --- | --- |
| T-piece − post-extubation | −0.8 | −2.3 – 0.6 |
| HFO−9.8 (40 L/min) − post-extubation | −1.2 | −1.7 – −0.2 |
| HFO−9.8 (60 L/min) − post-extubation | −1.0 | −1.8 – 0.3 |
| HFO−6.9 (40 L/min) − post-extubation | −0.7 | −1.1 – −0.4 |
| HFO−6.9 (60 L/min) − post-extubation | −0.6 | −1.7 – 0.6 |

PSV: pressure support ventilation; PEEP: positive end-expiratory pressure; HFO-9.8: HFO via endotracheal tube with a 9.8 mm expiratory port; HFO-6.9: HFO via endotracheal tube with a 6.9 mm expiratory port; ∆Pes: tidal swing of esophageal pressure during inspiration.

Data are presented as median. *p*-values represent global differences across PSV baseline, five study phases and post extubation determined by Friedman test. Post hoc pairwise comparisons were performed using Dunn’s test with Bonferroni correction. Paired differences were calculated as each study condition minus post-extubation ∆Pes. Values are median paired differences with 95% bootstrap confidence intervals (CI).

* P < 0.05 versus PSV Baseline.

† An additional Friedman comparison excluding PSV baseline showed no difference in ΔPes across T-piece, the four tracheal HFO conditions, and post-extubation measurements (*p* = 0.24)

**Table S6.** Physiological variables at baseline prior to each study phase

|  | Baseline 1 | Baseline 2 | Baseline 3 | Baseline 4 | Baseline 5 | *p*-value |
| --- | --- | --- | --- | --- | --- | --- |
| Respiratory rate, breaths/min | 20 (14–25) | 19 (14–24) | 19 (15–25) | 19 (14–25) | 20 (14–25) | 0.41 |
| Heart rate, beats/min | 91 (80–100) | 90 (72–107) | 87 (77–99) | 90 (80–104) | 89 (77–99) | 0.49 |
| Mean blood pressure, mm Hg | 92 (84–100) | 91 (83–105) | 94 (82–103) | 94 (88–105) | 93 (88–106) | 0.24 |
| SpO_2_, % | 99 (98–100) | 99 (98–100) | 99 (99–100) | 99 (99–100) | 99 (98–100) | 0.46 |
| EtCO_2_, mm Hg | 39 (32–43) | 38 (34–42) | 40 (33–43) | 40 (35–43) | 39 (34–43) | 0.72 |

EtCO_2_: end-tidal carbon dioxide pressure.

Data are presented as median (interquartile range). *p*-values represent global differences across five baseline phases determined by Friedman test.

**Table S7.** Mixed-effects sensitivity analyses for primary and secondary endpoints

|  | Treatment | Period/order | Previous treatment | HFO-9.8 (40 L/min) vs T-piece | HFO-9.8 (60 L/min) vs T-piece | HFO-6.9 (40 L/min) vs T-piece | HFO-6.9 (60 L/min) vs T-piece |
| --- | --- | --- | --- | --- | --- | --- | --- |
| Mean expiratory airway pressure | <0.001 | 0.32 | 0.28 | <0.001 | <0.001 | <0.001 | <0.001 |
| PEEP | <0.001 | 0.10 | 0.37 | <0.001 | <0.001 | <0.001 | <0.001 |
| ∆EELV_glob_ | <0.001 | 0.78 | 0.43 | <0.001 | <0.001 | <0.001 | <0.001 |
| V_T_ | 0.09 | 0.51 | 0.41 | 0.67 | 0.67 | 0.77 | 0.96 |
| Respiratory rate | <0.001 | 0.31 | 0.46 | 0.07 | 0.07 | 0.04 | <0.001 |
| ∆Pes | 0.99 | 0.10 | 0.46 | >0.99 | >0.99 | >0.99 | >0.99 |
| PTPes | 0.39 | 0.09 | 0.08 | >0.99 | >0.99 | >0.99 | 0.67 |
| Pmus | 0.88 | 0.07 | 0.33 | >0.99 | >0.99 | >0.99 | >0.99 |
| ∆P_L,dyn_ | <0.001 | 0.28 | 0.29 | 0.37 | 0.004 | <0.001 | <0.001 |
| EtCO_2_ | 0.07 | 0.10 | 0.08 | 0.32 | 0.35 | 0.48 | 0.52 |
| SpO_2_ | 0.71 | 0.92 | 0.79 | 0.82 | 0.82 | 0.82 | 0.82 |

HFO-9.8: HFO via endotracheal tube with a 9.8 mm expiratory port; HFO-6.9: HFO via endotracheal tube with a 6.9 mm expiratory port; PEEP: positive end-expiratory pressure; ∆EELV_glob_: global change of end-expiratory lung volume; V_T_: tidal volume; ∆Pes: tidal swing of esophageal pressure during inspiration; PTPes: esophageal pressure time product per minute; Pmus: pressure generated by respiratory muscle during inspiration; ∆P_L,dyn_: dynamic transpulmonary driving pressure; EtCO_2_: end-tidal carbon dioxide pressure.

Values are P values. Models included patient as a random intercept and treatment condition, study period/order, and preceding treatment condition as fixed effects. Pairwise comparisons versus T-piece were adjusted using the Holm method.

**Table S8** Bench–derived respiratory mechanics across flow rates and expiratory port diameters

| **PEEP, cm H_2_O** | | | | | | |
| --- | --- | --- | --- | --- | --- | --- |
| Flow rate | 10 L/min | 20 L/min | 30 L/min | 40 L/min | 50 L/min | 60 L/min |
| HFO–6.9 | 0.3 ± 0.1 | 0.9 ± 0.1 | 1.6 ± 0.2 | 2.8 ± 0.2 | 4.3 ± 0.2 | 6.0 ± 0.2 |
| HFO–8.6 | 0.2 ± 0.1 | 0.6 ± 0.1 | 1.1 ± 0.1 | 1.9 ± 0.1 | 2.9 ± 0.2 | 4.2 ± 0.2 |
| HFO–9.8 | 0.1 ± 0.1 | 0.3 ± 0.1 | 0.6 ± 0.1 | 1.0 ± 0.1 | 1.5 ± 0.1 | 2.2 ± 0.1 |
| HFO–14.6 | 0.1 ± 0.0 | 0.1 ± 0.1 | 0.3 ± 0.0 | 0.4 ± 0.1 | 0.7 ± 0.1 | 0.9 ± 0.1 |
| *Linear mixed–effects modeling demonstrated:*  Flow main effect, *p* < 0.001;  Diameter main effect, *p* < 0.001;  Flow × diameter interaction, *p* < 0.001. | | | | | | |
| **Peak inspiratory airway pressure, cm H₂O** | | | | | | |
| Flow rate | 10 L/min | 20 L/min | 30 L/min | 40 L/min | 50 L/min | 60 L/min |
| HFO–6.9 | –1.5 ± 1.1 | –1.2 ± 1.1 | –0.7 ± 1.1 | 0.1 ± 1.3 | 1.2 ± 1.4 | 2.7 ± 1.4 |
| HFO–8.6 | –1.6 ± 1.2 | –1.4 ± 1.2 | –1.1 ± 1.2 | –0.6 ± 1.2 | 0.2 ± 1.3 | 1.2 ± 1.4 |
| HFO–9.8 | –1.5 ± 1.1 | –1.4 ± 1.1 | –1.2 ± 1.1 | –0.9 ± 1.1 | –0.6 ± 1.1 | 0.0 ± 1.2 |
| HFO–14.6 | –1.5 ± 1.0 | –1.4 ± 1.0 | –1.4 ± 1.0 | –1.3 ± 1.0 | –1.2 ± 1.1 | –1.0 ± 1.0 |
| *Linear mixed–effects modeling demonstrated:*  Flow main effect, *p* < 0.001;  Diameter main effect, *p* < 0.001;  Flow × diameter interaction, *p* < 0.001. | | | | | | |
| **Mean inspiratory airway pressure, cm H₂O** | | | | | | |
| Flow rate | 10 L/min | 20 L/min | 30 L/min | 40 L/min | 50 L/min | 60 L/min |
| HFO–6.9 | –0.8 ± 0.7 | –0.5 ± 0.6 | 0.1 ± 0.7 | 1.0 ± 0.8 | 2.2 ± 0.9 | 3.8 ± 0.9 |
| HFO–8.6 | –0.9 ± 0.7 | –0.7 ± 0.7 | –0.3 ± 0.7 | 0.3 ± 0.8 | 1.1 ± 0.8 | 2.3 ± 0.9 |
| HFO–9.8 | –0.8 ± 0.6 | –0.7 ± 0.6 | –0.5 ± 0.6 | –0.2 ± 0.7 | 0.2 ± 0.7 | 0.8 ± 0.7 |
| HFO–14.6 | –0.8 ± 0.6 | –0.8 ± 0.6 | –0.7 ± 0.6 | –0.6 ± 0.6 | –0.5 ± 0.7 | –0.3 ± 0.6 |
| *Linear mixed–effects modeling demonstrated:*  Flow main effect, *p* < 0.001;  Diameter main effect, *p* < 0.001;  Flow × diameter interaction, *p* < 0.001. | | | | | | |
| **Peak expiratory airway pressure, cm H₂O** | | | | | | |
| Flow rate | 10 L/min | 20 L/min | 30 L/min | 40 L/min | 50 L/min | 60 L/min |
| HFO–6.9 | 1.4 ± 0.8 | 1.9 ± 0.8 | 2.7 ± 0.8 | 3.7 ± 0.8 | 5.3 ± 0.8 | 7.1 ± 0.8 |
| HFO–8.6 | 1.3 ± 0.8 | 1.7 ± 0.8 | 2.2 ± 0.8 | 3.0 ± 0.8 | 4.1 ± 0.9 | 5.3 ± 0.9 |
| HFO–9.8 | 1.1 ± 0.7 | 1.3 ± 0.7 | 1.6 ± 0.8 | 2.1 ± 0.8 | 2.6 ± 0.8 | 3.4 ± 0.8 |
| HFO–14.6 | 1.0 ± 0.7 | 1.1 ± 0.7 | 1.2 ± 0.7 | 1.4 ± 0.7 | 1.6 ± 0.7 | 1.9 ± 0.7 |
| *Linear mixed–effects modeling demonstrated:*  Flow main effect, *p* < 0.001;  Diameter main effect, *p* < 0.001;  Flow × diameter interaction, *p* < 0.001. | | | | | | |
| **Mean expiratory airway pressure, cm H₂O** | | | | | | |
| Flow rate | 10 L/min | 20 L/min | 30 L/min | 40 L/min | 50 L/min | 60 L/min |
| HFO–6.9 | 0.6 ± 0.3 | 1.2 ± 0.3 | 2.0 ± 0.3 | 3.1 ± 0.4 | 4.6 ± 0.4 | 6.4 ± 0.4 |
| HFO–8.6 | 0.5 ± 0.3 | 0.9 ± 0.3 | 1.5 ± 0.3 | 2.3 ± 0.3 | 3.3 ± 0.4 | 4.6 ± 0.4 |
| HFO–9.8 | 0.4 ± 0.2 | 0.6 ± 0.2 | 0.9 ± 0.3 | 1.3 ± 0.3 | 1.9 ± 0.3 | 2.6 ± 0.3 |
| HFO–14.6 | 0.3 ± 0.2 | 0.4 ± 0.2 | 0.5 ± 0.2 | 0.7 ± 0.2 | 0.9 ± 0.2 | 1.2 ± 0.2 |
| *Linear mixed–effects modeling demonstrated:*  Flow main effect, *p* < 0.001;  Diameter main effect, *p* < 0.001;  Flow × diameter interaction, *p* < 0.001. | | | | | | |
| **∆P_L,dyn_, cm H_2_O** | | | | | | |
| Flow rate | 10 L/min | 20 L/min | 30 L/min | 40 L/min | 50 L/min | 60 L/min |
| HFO–6.9 | 8.1 ± 3.2 | 7.8 ± 3.1 | 7.5 ± 3.0 | 7.2 ± 2.9 | 6.9 ± 2.8 | 6.6 ± 2.7 |
| HFO–8.6 | 8.1 ± 3.1 | 7.9 ± 3.1 | 7.7 ± 3.0 | 7.4 ± 2.9 | 7.2 ± 2.9 | 7.0 ± 2.7 |
| HFO–9.8 | 8.3 ± 3.1 | 8.2 ± 3.2 | 8.1 ± 3.2 | 8.0 ± 3.1 | 7.8 ± 3.0 | 7.7 ± 3.0 |
| HFO–14.6 | 8.4 ± 3.2 | 8.4 ± 3.2 | 8.3 ± 3.2 | 8.2 ± 3.2 | 8.1 ± 3.2 | 8.0 ± 3.2 |
| *Linear mixed–effects modeling demonstrated:*  Flow main effect, *p* < 0.001;  Diameter main effect, *p* < 0.001;  Flow × diameter interaction, *p* < 0.001. | | | | | | |
| **∆EELV, mL** | | | | | | |
| Flow rate | 10 L/min | 20 L/min | 30 L/min | 40 L/min | 50 L/min | 60 L/min |
| HFO–6.9 | 7 ± 3 | 15 ± 6 | 23 ± 9 | 32 ± 12 | 39 ± 15 | 47 ± 17 |
| HFO–8.6 | 7 ± 4 | 13 ± 5 | 19 ± 7 | 25 ± 9 | 32 ± 12 | 39 ± 15 |
| HFO–9.8 | 3 ± 2 | 5 ± 2 | 8 ± 3 | 12 ± 4 | 14 ± 5 | 19 ± 8 |
| HFO–14.6 | 1 ± 1 | 2 ± 1 | 4 ± 2 | 6 ± 2 | 8 ± 3 | 12 ± 4 |
| *Linear mixed–effects modeling demonstrated:*  Flow main effect, *p* < 0.001;  Diameter main effect, *p* < 0.001;  Flow × diameter interaction, *p* < 0.001. | | | | | | |
| **V_T_, mL** | | | | | | |
| Flow rate | 10 L/min | 20 L/min | 30 L/min | 40 L/min | 50 L/min | 60 L/min |
| HFO–6.9 | 201 ± 96 | 194 ± 94 | 185 ± 90 | 176 ± 85 | 168 ± 80 | 161 ± 77 |
| HFO–8.6 | 203 ± 97 | 197 ± 95 | 190 ± 91 | 184 ± 89 | 177 ± 85 | 172 ± 82 |
| HFO–9.8 | 208 ± 100 | 206 ± 99 | 202 ± 98 | 198 ± 96 | 194 ± 94 | 191 ± 91 |
| HFO–14.6 | 211 ± 102 | 210 ± 101 | 208 ± 101 | 205 ± 101 | 202 ± 99 | 199 ± 99 |
| *Linear mixed–effects modeling demonstrated:*  Flow main effect, *p* < 0.001;  Diameter main effect, *p* < 0.001;  Flow × diameter interaction, *p* < 0.001. | | | | | | |
| **Peak inspiratory flow, L/s** | | | | | | |
| Flow rate | 10 L/min | 20 L/min | 30 L/min | 40 L/min | 50 L/min | 60 L/min |
| HFO–6.9 | 0.38 ± 0.16 | 0.37 ± 0.16 | 0.35 ± 0.16 | 0.33 ± 0.15 | 0.32 ± 0.14 | 0.30 ± 0.13 |
| HFO–8.6 | 0.38 ± 0.16 | 0.37 ± 0.16 | 0.35 ± 0.15 | 0.34 ± 0.15 | 0.33 ± 0.14 | 0.32 ± 0.14 |
| HFO–9.8 | 0.39 ± 0.17 | 0.38 ± 0.17 | 0.38 ± 0.17 | 0.37 ± 0.16 | 0.36 ± 0.16 | 0.36 ± 0.16 |
| HFO–14.6 | 0.39 ± 0.17 | 0.39 ± 0.17 | 0.39 ± 0.17 | 0.38 ± 0.17 | 0.38 ± 0.17 | 0.37 ± 0.17 |
| *Linear mixed–effects modeling demonstrated:*  Flow main effect, *p* < 0.001;  Diameter main effect, *p* < 0.001;  Flow × diameter interaction, *p* < 0.001. | | | | | | |
| **Peak expiratory flow, L/s** | | | | | | |
| Flow rate | 10 L/min | 20 L/min | 30 L/min | 40 L/min | 50 L/min | 60 L/min |
| HFO–6.9 | 0.26 ± 0.12 | 0.25 ± 0.12 | 0.24 ± 0.11 | 0.22 ± 0.11 | 0.21 ± 0.10 | 0.20 ± 0.09 |
| HFO–8.6 | 0.26 ± 0.12 | 0.25 ± 0.12 | 0.24 ± 0.11 | 0.23 ± 0.11 | 0.22 ± 0.10 | 0.21 ± 0.10 |
| HFO–9.8 | 0.27 ± 0.13 | 0.27 ± 0.13 | 0.26 ± 0.13 | 0.25 ± 0.12 | 0.24 ± 0.12 | 0.24 ± 0.11 |
| HFO–14.6 | 0.28 ± 0.14 | 0.28 ± 0.14 | 0.28 ± 0.14 | 0.27 ± 0.14 | 0.27 ± 0.13 | 0.26 ± 0.13 |
| *Linear mixed–effects modeling demonstrated:*  Flow main effect, *p* < 0.001;  Diameter main effect, *p* < 0.001;  Flow × diameter interaction, *p* < 0.001. | | | | | | |
| **Inspiratory airway resistance, cm H₂O s/L** | | | | | | |
| Flow rate | 10 L/min | 20 L/min | 30 L/min | 40 L/min | 50 L/min | 60 L/min |
| HFO–6.9 | 15.4 ± 4.6 | 16.1 ± 4.6 | 16.7 ± 4.6 | 17.5 ± 4.9 | 18.4 ± 5.3 | 19.1 ± 5.6 |
| HFO–8.6 | 15.1 ± 4.6 | 15.7 ± 4.7 | 16.2 ± 4.7 | 16.9 ± 4.8 | 17.5 ± 5.1 | 18.0 ± 5.4 |
| HFO–9.8 | 14.7 ± 4.5 | 14.9 ± 4.6 | 15.3 ± 4.6 | 15.6 ± 4.6 | 15.7 ± 4.7 | 16.3 ± 4.8 |
| HFO–14.6 | 14.3 ± 4.4 | 14.7 ± 4.5 | 15.0 ± 4.5 | 15.1 ± 4.5 | 15.5 ± 4.7 | 15.8 ± 4.6 |
| *Linear mixed–effects modeling demonstrated:*  Flow main effect, *p* < 0.001;  Diameter main effect, *p* < 0.001;  Flow × diameter interaction, *p* < 0.001. | | | | | | |
| **Expiratory airway resistance, cm H₂O s/L** | | | | | | |
| Flow rate | 10 L/min | 20 L/min | 30 L/min | 40 L/min | 50 L/min | 60 L/min |
| HFO–6.9 | 14.4 ± 4.9 | 15.3 ± 4.9 | 15.9 ± 4.6 | 16.7 ± 4.7 | 17.6 ± 4.6 | 18.2 ± 4.2 |
| HFO–8.6 | 14.1 ± 4.8 | 14.7 ± 4.8 | 15.1 ± 4.8 | 15.8 ± 4.5 | 16.5 ± 4.4 | 17.1 ± 4.8 |
| HFO–9.8 | 13.6 ± 4.7 | 14.2 ± 4.5 | 14.4 ± 4.8 | 14.6 ± 4.6 | 15.3 ± 4.6 | 15.7 ± 4.6 |
| HFO–14.6 | 12.9 ± 4.6 | 13.3 ± 4.8 | 13.7 ± 4.9 | 13.9 ± 4.9 | 14.5 ± 4.8 | 15.0 ± 4.6 |
| *Linear mixed–effects modeling demonstrated:*  Flow main effect, *p* < 0.001;  Diameter main effect, *p* < 0.001;  Flow × diameter interaction, *p* < 0.001. | | | | | | |

PEEP: positive end–expiratory pressure; HFO-6.9: HFO via endotracheal tube with a 6.9 mm expiratory port; HFO-8.6: HFO via endotracheal tube with a 8.6 mm expiratory port; HFO-9.8: HFO via endotracheal tube with a 9.8 mm expiratory port; HFO-14.6: HFO via endotracheal tube with a 14.6 mm expiratory port; ∆P_L,dyn_: dynamic transpulmonary driving pressure; ∆EELV: change of end-expiratory lung volume; V_T_: tidal volume.

Values are presented as mean ± SD. Flow rate and inner diameter were analyzed using linear mixed–effects models, with simulated scenarios modeled as random intercepts. *p*-values correspond to fixed effects derived from the model.

Inspiratory airway resistance could not be determined in 4 simulated scenarios and expiratory airway resistance in 12 scenarios due to peak flow < 0.2 L/s. Analyses for these variables were therefore restricted to simulations in which peak flow met the measurement threshold.

**Table S9** Summary of physiological differences between HFO via endotracheal tube and T–piece SBT stratified by interface diameter

| Physiological variable^*^ | HFO–9.8 | HFO–6.9 |
| --- | --- | --- |
| Airway pressure | √ | √ |
| ΔEELV | √ | √ |
| Dependent fraction of ventilation | × | √ |
| GI index | × | √ |
| PaO_2_/FiO_2_ | × | √ |
| ΔP_L,dyn_ | √ | √ |
| WOB_ela_ | × | √ |
| Respiratory rate | × | √ |
| Minute ventilation | × | √ |
| Peak flow | √ | √ |
| Airway resistance | √ | √ |
| PaCO_2_ and EtCO_2_ | × | × |
| V_T_ | × | × |
| Hemodynamics parameters | × | × |
| ∆Pes, PTPes, Pmus and WOB | × | × |

HFO-9.8: HFO via endotracheal tube with a 9.8 mm expiratory port; HFO-6.9: HFO via endotracheal tube with a 6.9 mm expiratory port; ∆EELV: change of end–expiratory lung volume; GI index: global inhomogeneity index; ∆P_L,dyn_: dynamic transpulmonary driving pressure; WOB_ela_: elastic work of breathing; EtCO_2_: end-tidal carbon dioxide pressure; V_T_: tidal volume; ∆Pes: tidal swing of esophageal pressure during inspiration; PTPes: esophageal pressure time product per minute; Pmus: pressure generated by respiratory muscle during inspiration; WOB: work of breathing.

^*^Physiological variables are summarized according to whether a statistically significant difference was observed between HFO via endotracheal tube and T-piece SBT. A check mark (√) indicates a significant difference, whereas a cross (×) indicates no significant difference. A variable was considered different if a significant difference was observed at any tested flow rate.

**Figures**

**Figure S1.** Two types of high-flow oxygen via endotracheal tube interfaces used in the clinical study. (A) The tracheal HFO interface connects to the endotracheal tube. (B) Comparison of two interfaces with different expiratory port diameters. The upper and lower arrows point to the narrowest expiratory ports of interface of 9.8 mm and 6.9 mm, respectively. (C) and (D) The narrowest expiratory inner diameter of the two interface types is marked.

**Figure S2.** Representative respiratory waveforms across the five study phases. Rpresentative tracings of flow, airway pressure, esophageal pressure, and transpulmonary pressure obtained from one patient during the five study phases. For each condition, three consecutive breaths were selected. Because the duration of the selected breaths varied slightly across conditions, the time axis was rescaled to a uniform format (2 s per division) to facilitate visual comparison. These tracings are presented for illustrative purposes only and are not intended for quantitative analysis. HFO-9.8: HFO via endotracheal tube with a 9.8 mm expiratory port; HFO-6.9: HFO via endotracheal tube with a 6.9 mm expiratory port.

**Figure S3.** Four types of high-flow oxygen via endotracheal tube interfaces used in the bench experiment. (A-D) Tracheal HFO interfaces with minimal internal expiratory port diameters of 6.9 mm, 8.6 mm, 9.8 mm, and 14.6 mm, respectively. The expiratory port diameter of each interface was measured at the narrowest expiratory outlet using a digital caliper after zero calibration.

**Figure S4.** Exploratory subgroup plots according to admission category. Individual values and medians are shown for mean expiratory airway pressure, PEEP, ∆EELV, and ∆P_L,dyn_ across the five SBT conditions, stratified by septic shock (n=8), pneumonia (n=8), and stroke (n=4). These variables were selected to illustrate pressure generation and downstream lung-volume/stress responses related to the pressure-threshold effect of tracheal HFO. No formal subgroup hypothesis testing was performed because subgroup sizes were small. HFO-9.8: HFO via endotracheal tube with a 9.8 mm expiratory port; HFO-6.9: HFO via endotracheal tube with a 6.9 mm expiratory port; PEEP: positive end-expiratory pressure; ∆EELV: change of end–expiratory lung volume; ∆P_L,dyn_: dynamic transpulmonary driving pressure.
